# Novel and multiple targets for chimeric antigen receptor-based therapies in lymphoma

**DOI:** 10.3389/fonc.2024.1396395

**Published:** 2024-04-22

**Authors:** Yifan Pang, Nilanjan Ghosh

**Affiliations:** Department of Hematologic Oncology and Blood Disorders, Atrium Health Levine Cancer Institute, Wake Forest School of Medicine, Charlotte, NC, United States

**Keywords:** chimeric antigen receptor, B-cell lymphoma, Hodgkin lymphoma, T-cell lymphoma, adoptive cellular immunotherapy

## Abstract

Chimeric antigen receptor (CAR) T-cell therapy targeting CD19 in B-cell non-Hodgkin lymphoma (NHL) validates the utility of CAR-based therapy for lymphomatous malignancies. Despite the success, treatment failure due to CD19 antigen loss, mutation, or down-regulation remains the main obstacle to cure. On-target, off-tumor effect of CD19-CAR T leads to side effects such as prolonged B-cell aplasia, limiting the application of therapy in indolent diseases such as chronic lymphocytic leukemia (CLL). Alternative CAR targets and multi-specific CAR are potential solutions to improving cellular therapy outcomes in B-NHL. For Hodgkin lymphoma and T-cell lymphoma, several cell surface antigens have been studied as CAR targets, some of which already showed promising results in clinical trials. Some antigens are expressed by different lymphomas and could be used for designing tumor-agnostic CAR. Here, we reviewed the antigens that have been studied for novel CAR-based therapies, as well as CARs designed to target two or more antigens in the treatment of lymphoma.

## Introduction

CARs are synthetic molecules that are encoded by an antigen-binding domain – typically a monoclonal antibody-based single-chain fragment variable (scFv), an extracellular hinge to improve immune synapses formation, a transmembrane anchor, a costimulatory and intracellular domain for signal transduction (1). Once expressed by the transduced cells, most commonly T-cells, sometimes NK-cells, CARs improve the homing of T or NK cells to tumor to facilitate and enhance tumor-specific killing. Not all tumor antigens can become CAR targets, only the surface antigens with high densities can be recognized by CAR and fully activate modified immune cells (2, 3).

## B-cell non-Hodgkin lymphoma

A rapidly growing list of indications underscores the success of CD19-targeted CAR T-cell therapy in B-cell non-Hodgkin lymphoma (NHL). Durable complete remission has been consistently reported in clinical trials utilizing CD19-CAR T in relapsed/refractory (R/R) B-NHL, indicating the curative potential of CD19-CAR T (4). However, for the more than 50% of patients who are refractory to or relapse after CD19-CAR T, there is an urgent need for alternative therapeutic options (1). The causes of resistance can be loosely divided into three categories: patient factors, CAR design factors, and target antigen modulation. Examples of patient factors include high disease burden, microbiome, and baseline cytokine milieu (5). CAR design factors, including transmembrane domain architecture, costimulatory domain, immune synapse spacing, affinity to antigen, are vital to the efficacy and safety of CAR T (1). For instance, high affinity of the CAR to CD19 antigen may not improve efficacy but promote T-cell exhaustion instead. Michelozzi, et al., recently demonstrated that low-affinity CD19-CAR T had enhanced *in vivo* expansion, prolonged persistence, and better tolerability, than traditional high-affinity CD19-CAR T (6). CAR integration using non-retroviral methods, such as using adeno-associated virus or non-viral gene editing to transfer a CD19-CAR into the T-cell receptor alpha constant (TRAC) region, has demonstrated improved functionality compared to traditional retroviral transduction (7). A novel TRAC-integrated CD19-targeted CAR T product is currently undergoing a phase 1 clinical trial in patients with R/R large B-cell lymphoma (NCT05757700). Target antigen modulation is one of the most common mechanisms of resistance. In different studies, CD19 antigen loss or down-regulation was seen in 25% to 33% of the relapsed cases (8–10). CAR-based therapies targeting other B-cell antigens hold the promise to improve the outcome of patients with R/R B-NHL. Active clinical trials of novel CAR-based therapy in B-NHL are listed in **Table 1**.

**Table 1:** Active clinical trials of novel CAR therapy for B-cell non-Hodgkin Lymphoma.

CAR Target	NCT number	Phase	Location	Notes
CD20	NCT03277729	1 and 2	US - Fred Hutch	CNS involvement eligible
	NCT05360238	1 and 2	US - multicenter	Cell name: MB-106
	NCT05784441	1	US - multicenter	Cell name: JNJ-90009530
CD22	NCT05972720	2	US - multicenter	Prior successful phase 1 NCT04088890 yielded the RP2D of 1 million cells/kg
	NCT04571138	1 and 2	US - multicenter	In both B-ALL and B-NHL
κLC	NCT04223765	1	US - UNC	B-NHL and CLL/SLL
	NCT00881920	1	US - Baylor	B-NHL, CLL, myeloma
CD79b	NCT05773040	1	US - MDACC	Cell-name: JV213
	NCT05312476	2	China	Inducible caspace-9 gene system
CD70	NCT05948033	1 and 2	China	Requires ≥ 10% CD70 antigen expression
ROR1	NCT05694364	1	US - Moffitt	PD-1 switchable
	NCT05588440	1 and 2	US - multicenter	Highly specific anti-ROR1 scFv with no preclinical evidence of on-target off-tumor toxicity
BAFF	NCT05312801	1	US - Cleveland	
CD19/CD20	NCT05149391	1	China	Cell name: C-CAR039
	NCT04007029	1	US - UCLA	
	NCT04186520	1 and 2	US - MCW	flexible manufacturing schema
	NCT04697940	1 and 2	China	decitabine-primed
	NCT05797233	1	US - NCI	
	NCT04989803	1	US - multicenter	Cell name: KITE-363 or KITE-753
	NCT06014762	1	US - multicenter	Allo-CAR with Rimiducid to reduce neurotoxicity
	NCT05826535	1 and 2	US - multicenter	Cell name: IMPT-314
	NCT05421663	1	US - multicenter	
	NCT04792489	2	US - multicenter	Cell name: MB-CART2019.1
CD19/CD22	NCT05091541	1 and 2	China	
	NCT05651100	1 and 2	China	Sequential CD19 and CD22 CAR T instead of bispecific CAR T
	NCT06005649	1 and 2	China	
	NCT03241940	1	US - Stanford	
	NCT03233854	1	US - Stanford	CAR T in combination with chemotherapy NKTR-255, CNS involvement eligible
	NCT05098613	1	US - University of Colorado	
	NCT03448393	1	US - NCI	All B-cell cancer in children and young adults
CD19/BCMA	NCT06097455	1	Spain	Cell name: ARI0003
CD19/CD70	NCT05436496	1 and 2	China	Sequential CD19 and CD70 CAR T instead of bispecific CAR T
CD20/CD22	NCT05607420	1 and 2	US - multicenter	Cell name: UCART20x22; Allo-CAR
CD20/CD79a	NCT05169489	1 and 2	US - multicenter	Cell name: bbT369
CD19/CD20/ CD22	NCT05418088	1	US - OSU	

US, United States; Fred Hutch, Fred Hutchinson Cancer Center; CNS, central nervous system; RP2D, recommended phase 2 dose; ALL, acute lymphoblastic leukemia; NHL, non-Hodgkin Lymphoma; UNC, University of North Carolina; MDACC, MD Anderson Cancer Center; UCLA, University of California, Los Angeles; MCW, Medical College of Wisconsin; NCI, National Cancer Institute; OSU, Ohio State University.

### CD20

Anti-CD20 monoclonal antibody revolutionized the treatment of B-cell NHL and greatly improved patient outcomes. CD20 theoretically can elicit a more robust T-cell activation than CD19 because of slower endocytosis and stronger immunological synapses formation (11). However, the concern of developing resistance after recurrent rituximab exposure may have played a role in the delay in clinical development of CD20-CAR therapy (12). Reassuringly, CD20 antigen loss or mutation is uncommon in relapsed B- NHL and is not a significant cause of treatment failure (13, 14). The success of bispecific CD20 and CD3 T-cell engagers further confirm the utility of CD20-targeting in R/R B-NHL (15). *In vitro* studies demonstrated preserved cytotoxicity of CD20-CAR T in CD20-downregulated cancers (3).

Till, et al., treated seven patients with R/R follicular lymphoma (FL) or mantle cell lymphoma (MCL) with first generation CD20-CAR T (16). All patients had previous rituximab exposure. Responses included two complete remissions (CR) and three partial responses (PR). None had adverse events related to T-cell infusions. Later, Till, et al., treated three patients with a third generation CD20-CAR T with both CD28 and 4-1BB costimulatory domains, two achieved durable CR (17). Another third generation CD20-CAR T, MB-106, showed efficacy and tolerability in a single center clinical trial (NCT03277729) (18, 19). In 16 patients (12 FL, 2 MCL, 1 CLL, 1 diffuse large B-cell lymphoma), overall response rate (ORR) was 94%, CR 62%, and 90% of the CR was durable (duration of response, 3-18 months); no grade ≥ 3 cytokine release syndrome (CRS) or immune effector cell-associated neurotoxicity syndrome (ICANS) was observed. MB-106 is currently being tested in a multicenter phase 1/2 trial (NCT05360238) (20). Several other CD20-CAR Ts have also entered clinical trials for R/R B-NHL. C-CAR066 was previously tested in a phase 1 clinical trial in China. Fourteen patients were treated, including 11 with diffuse large B-cell lymphoma (DLBCL) and three with transformed FL; 12 previously received CD19-CAR T, one received bispecific CD19/20-CAR T and one received bispecific CD19/79b-CAR T. ORR was 92.9% and CR was achieved in 57.1%. Four remained in CR after 24 months. Grade ≥3 CRS only occurred in one patient and none experienced ICANS (21). C-CAR066 recently entered multicenter phase 1 studies in the US (NCT05784441). MB-CART20.1 is another CD20-CAR T which was tested in a Germany multicenter phase 1 trial (22). Durable CR was seen in 3 of the 10 treated patients. No dose-limiting toxicity (DLT) occurred in the trial, but the trial was stopped early due to COVID-19.

### CD22

CD22 is a B-cell-specific transmembrane glycoprotein involved in B-cell survival, proliferation and function (23). Anti-CD22 antibody-drug conjugates (ADC) inotuzumab ozogamicin and moxetumomab pasudotox, were approved for B-cell acute lymphoblastic leukemia (ALL) and hairy cell leukemia, respectively (24, 25). Despite strong CD22 expression on mature B-cells, naked anti-CD22 antibody and ADC all failed to demonstrate significant benefit over standard of care chemoimmunotherapy in patients with CD22+ R/R B-NHL (26, 27). The challenges of CD22-targeted therapy in B-NHL include the higher variability of CD22 expression on lymphoma cells than CD19 and CD20, down regulation of CD22 and low antigen density (28, 29). CD22 has a bulky extracellular structure, making it difficult to target (30).

With the success of anti-CD22 ADC, CD22-CAR T was first developed for B-ALL. Haso, et al., found that CAR targeting the proximal domain of CD22 yielded superior antileukemic activity in preclinical models (31). Proximal-targeting CD22-CAR T was then tested in a phase 1 study in 21 children and adults with R/R B-ALL (32). Potent efficacy was seen even in patients with CD19^dim^ or CD19-negative disease. Currently, various CD22-CAR T have entered phase 1 and/or 2 clinical trials, but no phase 3. In a meta-analysis with a data cutoff in March 2022, a total of seven CD22-CAR T clinical trials with 149 patients had primary efficacy data, but only two enrolled patients with lymphoma (33). A single center phase 1/1b study at Stanford University (NCT04088890) is one of the first trials using autologous CD22-CAR T to treat R/R LBCL (34, 35). Among 41 enrolled patients, 40 underwent leukapheresis and 38 (95%) had successful manufacturing of cells. Twenty-nine patients were treated at dose level (DL)1 (1 x 10^6^/kg) and 9 at DL2 (3 x 10^6^/kg); all but 1 progressed after prior CART19. ORR was 68%. Twenty (53%) entered CR. Response was similar between DL1 and DL2. At a median follow up of 18.4 months (range, 1.5-38.6), nineteen patients remained in CR. Median progression-free survival (PFS) was 2.9 months (range, 1.7-NR) and overall survival (OS) 22.5 months (range, 8.3-NR). CD22-CAR T was well tolerated especially at DL1, and proceeded to a phase 2 clinical trial (NCT05972720) with DL1 as the recommended phase 2 dose (RP2D) (35). Another CD22-CAR T, SCRI-CAR22v2, is being tested in a phase 1/2 multicenter clinical trial, PLAT-07, in pediatric and young adult patients with R/R CD22+ leukemia or lymphoma (NCT04571138). SCRI-CAR22v2 is an improved version of its predecessor SCRI-CAR22v1, with a shorter linker and transmembrane region, and better activity and survival than the latter (36). Allogeneic CD22-CAR T (alloCART22) has been developed. In preclinical models, alloCART22 demonstrated pharmacologic activity against CD22+ tumor and successful evasion of host innate and adaptive immune rejection (37). The safety and efficacy of alloCART22 are yet to be evaluated by clinical trials.

Hemophagocytic lymphohistiocytosis (HLH), a subtype of severe cytokine release syndrome, is associated with higher disease burden, pre-infusion NK-cell lymphopenia and persistent elevation of HLH-related cytokines including IFNγ, IL-1β and IL-18. HLH more common in patients receiving CD22-CAR T (35.6%) than CD19-CAR T (14.8%) (38, 39). On the other hand, ICANS is less common and less severe with CD22-CAR T, likely due to different cytokine milieu, and absence of CD22 expression on the blood brain barrier and oligodendrocyte precursor cells (40).

High peak CD22-CAR T expansion and high expression of the activator protein-1 Fos/Jun are associated with response and toxicity to CD22-CAR T (41). Fos/Jun heterodimer are important transcription factors in activated T-cells and enhances the transcription of inflammatory cytokines such as IL-2 (42). CD22-CAR T from patients who progressed do not have high Fos/Jun, instead, have higher proportion of terminal effector memory cells and higher expression levels of immunomodulatory killer cell immunoglobulin like receptors (41). Another main cause of relapse remains CD22 antigen loss (35, 43). Unlike CD19 antigen loss which is usually mediated by mutation and alternative splicing, down-regulation of CD22 on cell surface makes cells resistant to CD22 targeting (32). Upregulation of CD22 expression by bryostatin-1 pretreatment could potentially re-sensitize lymphoma cells to CD22-CAR T (44). Another major development to overcome antigen loss is bispecific or multi-specific CARs, which will be discussed in detail in later sections.

The natural ligand of CD22 is a cell surface trisaccharide (45). Transferring natural ligand-mimicking CD22-specific polysaccharide onto cell surface is a novel way of designing adoptive cell therapy. Wang, et al., designed CD22-targeting NK-cells by glycoengineering, making NK-cells present a modified polysaccharide ligand on their surface. Not strictly a CAR-NK, the engineered NK-cells could effectively bind to CD22-positive lymphoma cells and exert cytotoxicity in preclinical models (46). Traditional CAR-NKs expressing CD22-scFv have also been studied preclinically (47, 48).

### CD19/CD20

Bispecific CAR T targeting two B-cell antigens can be activated upon binding with either antigens or both, thereby enhancing inflammatory cytokine production, and reducing resistance from single antigenic loss (49). Tandem CD19/20-CAR T targets both CD19 and CD20 antigens with a single CAR vector; the CD20 and CD19 binding domains are hinged with a flexible linker. Preclinical data of tandem CD19/20-CAR T revealed several important findings (50). First, CD19-scFv should be placed proximally and CD20-scFv distally to T-cell membrane, to allow optimal conformation of binding. Second, most of the cytokine release after CD19/20-CAR T is likely driven by CD20 recognition. Thirdly, tandem CD19/20-CAR T was more effective than a combination of CD19- and CD20-CAR T.

Based on supportive preclinical data, Shah, et al., conducted a first-in-human trial of bispecific CD19/20-CAR T, LV20.19 (MB CART2019.1, or zamtocabtagene autoleucel, zamto-cel) in patents with R/R B-NHL (51). In the first 22 patients treated, 18 (82%) responded at day 28, including 14 (64%) CR. Rates of grade 3-4 CRS and ICANS were 5% and 14%, respectively. For the patients who received a target dose of 2.5 million cells/kg (n=16), two-year PFS and OS were 44% and 69% at a median follow up of 31 months (range, 2-40) (52).

R/R MCL remains a challenging clinical entity, especially those cases with post-BTKi relapse and/or TP53 aberration. Tandem CD19/20-CAR T showed encouraging results in this hard-to-treat population. In a phase 1/2 clinical, 17 patients with R/R MCL received LV20.19, including 13 BTKi-refractory and seven TP53-mutated patients. The phase 2 portion utilized an adaptive manufacturing process to enhance the percentage of memory T-cells in the product. All patients responded at day 90, including 92% CR. Infection-related death occurred in two patients. Grade 3-4 CRS and ICANS occurred in zero and two patients, respectively. One-year PFS and OS rates were 77% and 84% (53).

The DALY I trial was a phase 1 clinical trial of zamto-cel in patients with R/R B-NHL (54). Twelve patients were enrolled and six received the recommended dose of 2.5 million cells/kg. ORR was 75% in the entire cohort and 5 patients achieved CR. Promisingly, all CRs were durable at 2-year follow up. No grade ≥ 3 CRS or ICANS were observed. Zamto-cel is currently undergoing phase 2 clinical trials, one as a 2^nd^ line therapy for patients with R/R B-NHL who are transplant-ineligible (DALY 2-EU), and the other for patients with R/R DLBCL after ≥ 2 prior lines of systemic therapy (DALY II USA) (55, 56). The interim analysis for DALY II USA demonstrated good efficacy and tolerability of zamto-cel (56). In 22 evaluable patients, CR rate was 46% and PR 36%, 6-month PFS was 64%. The treatment was well-tolerated, only two had transient and reversible grade 3 ICANS and none had grade ≥ 3 CRS.

Other tandem CD19/20-CAR Ts have been developed and undergone early phase clinical trials. Tong, et al., designed several CD19/20-CAR T. They found that one of the constructs, TanCAR7, had the strongest immunological synapse formation and the most potent antitumor activity (57). TanCAR7 was subsequently studied in a phase 1/2a clinical trial for heavily pretreated R/R B-NHL (NCT03097770). Among 99 enrolled patients, 92 underwent leukapheresis, and 87 received TanCAR7 (58). Twelve (14%) had previous autologous stem cell transplant (SCT) and 9 (10%) had previous exposure to CD19-CAR T. Best ORR was 78% and 70% had CR. Median PFS was 27.6 months (95%CI, 11 months to not reached), and 76% of the responses were durable beyond 12-months. Majority of subjects (70%) developed CRS, including 8 (9%) with grade 3 and 1 (1%) with grade 4, ICANS grade ≥ 3 occurred in 2 (2%) patients. Three patients had treatment-related mortality due to pneumonia or CRS-induced lung injury. CAR expansion was associated with response, but CAR persistence did not significantly impact duration of response (DoR). C-CAR039 is another CD19/20-CAR T that underwent phase 1 study at multiple sites in China (59). Forty-eight patients received C-CAR039, including 44 with LBCL, 3 with FL and 1 with MCL. While the percentage of patients who had prior CD19-CAR T was unknown, while 8 (16.7%) had prior autologous SCT. Response to C-CAR039 was exceptionally high, with ORR 91.5% and CR 85.1%. Estimated 2-year PFS was 66% (95%CI, 53.2 – 81.9%) and OS 77.9% (95%CI, 66.6-91.1%). Although treatment was well tolerated with low rates of grade 3 CRS and no grade 3 ICANS, three patients developed secondary malignancy, including 2 AML and 1 T-cell lymphoma (CAR transgene negative) (60). C-CAR039 is undergoing a multicenter phase 1 study in the US (NCT05421663).

Naïve and memory T-cells-enrichment may improve *in vivo* CAR T function by reducing cell exhaustion and improving persistence (61–63). Regulatory T-cells (Treg, marked by CD25) and myeloid cells (marked by CD14) may cause immunosuppression and reduce CAR T function (64, 65). Larson, et al., conducted a phase 1 trial using autologous naïve and memory T (TN/MEM)-selected, Treg and myeloid cell depleted, tandem CD19/20-CAR T to treat R/R B-NHL (63). Among 17 patients screened, 10 received infusion, yielding a CR rate of 70%. At 17-month follow up, median PFS and OS were not reached. The therapy was safe, no neurotoxicity or grade > 1 CRS were seen. Relapse could be re-treated with CART19/20.

The antigen recognition process of tandem CAR is theoretically unpredictable, therefore bicistronic CAR-T, placing CD20 and CD19 CAR separately on the T-cell, is another approach. Bicistronic CD19/20-CAR T has been successfully manufactured and showed preclinical efficacy in CD19-negative or CD20-negative B-cell lymphoma (66). A phase 1 clinical trial testing this product in R/R B-NHL is under way (NCT05797233).

Although less effective than tandem CD19/20-CAR T in preclinical studies, sequential CD19-CAR T and CD20-CAR T may be easier to manufacture. Sequential infusion strategy was tested in a pilot trial in China (67). In this study, 21 patients with R/R DLBCL who received CD19-CAR T but had undetectable circulating cell levels received CD20-CAR T to prevent relapse. Median interval between cell infusions was 3.72 months (range, 2.56-9). Subsequent CD20-CAR T was well tolerated, with low risk and severity of CRS and ICANS, echoing the observation in other CD20-CAR T studies. Durable CR was seen in 15 (71.4%) patients at a median follow up of 24.7 months (range, 11.64-45.86). CD20-CAR T consolidation post-CD19-CAR T may be a valuable strategy for patients with high-risk DLBCL.

### CD19/CD22

Dual CD19/CD22 targeting CAR T-cell therapy is a feasible method to bypass resistant mechanisms including antigen loss/mutation or down-regulation in B-cell malignancies. In B-ALL, CD19/22-CAR T showed good efficacy and DoR, but whether CD19/22-CAR T is better than CD19-CAR T alone remains debatable (68–70). Various strategies aiming to target both CD19 and CD22 have been developed, including CAR T cocktail, bicistronic or tandem CAR, some have entered clinical trials. Bispecific CD19/22-CAR-NK has proof-of-concept preclinical results (71).

CAR-T cocktail is a method of delivering multi-antigen targeting CAR-T cells based on the tumor’s antigen expression. CAR-T cocktail could be made by transducing autologous T-cells with two lentiviral vectors at the same time (dual transduction) or separately, or give single antigen-specific CAR T sequentially. Gardner, et al., at the Seattle Children’s Research Institute used lentiviral vectors to transduce either CD19 or CD22 CAR into autologous T-cells, resulting in pooled CD19-CAR T, CD22-CAR T, and cells with both CARs (72). However, early phase clinical trials using this pooled product in pediatric B-ALL demonstrated imbalanced CD19- and CD22-CAR T persistence, leading to antigen-negative relapse (73, 74). The Geno-Immune Medical Institute in China designed an autologous CAR-T platform, 4SCAR2.0, using an apoptosis-inducible intracellular domain CD28/CD27/CD3ζ-iCasp9 and variable extracellular domain (anti-CD19, CD11, CD30, CD70, etc) based on tumor antigen. 4SCAR2.0 is currently undergoing a multicenter clinical trial in China (NCT03125577). Each patient can receive multiple 4SCAR2.0 infusions that contains a single-targeting or dual-targeting CAR T. Preliminary results were available from 5 patients with refractory B-NHL, including 1 with PMBCL, 2 with DLBCL and 3 with FL. All received at least one infusion of 4SCAR-19 + 22, demonstrated durable remission, no ICANS and CRS as high as grade 1 (75, 76). It is plausible that repeated dosing improved the response rate and DoR, and low cell dose and the apoptosis-inducible domain reduced toxicity.

Sequential administration of CD19- and CD22-CAR T to treat R/R B-cell malignancies has been tested in China. In pilot study, Wang, et al., enrolled 89 patients, including 38 with R/R B-NHL and the rest with ALL. For the B-NHL cohort, each patient received around 5 x 10^6^ cells/kg of CD19- and CD22-CAR T on successive days. ORR was 72.2% and CR rate 50%, median DoR was 15 months (range, 12-17). At a median follow up of 14.4 months, the median PFS was 9.9 months (95%CI, 3.3-NR) and median OS 18.0 months (95%CI 6.1-NR) (77). The group subsequently conducted another clinical trial, sequentially administering high-dose chemotherapy followed by autologous SCT, CD22- and CD19-CAR T to patients with R/R B-NHL (78). In 49 consented patients, 42 completed sequential cellular therapy, including 23 with progressive disease and 9 with stable disease prior to SCT. Engraftment was not significantly impacted by post-SCT CAR-T. CRS was common (90%) but only 2 (5%) had grade ≥ 3 CRS. ICANS occurred in 9 (21%) and grade ≥ 3 in 2 (5%). High durable remission rates were reported, with 12-month PFS 95.7% (95%CI, 70.9-93.3%), 12-month OS 90.5% (95%CI. 76.6-96.3%), but those failed to respond by 3-month (n=4) had a dismal prognosis. Although response rate and survival appeared better than the previous study without SCT, the benefit of SCT should be carefully evaluated due to the financial toxicity of combination cellular therapy, and inherited risks of transplant such as hematologic toxicity, organ toxicity, secondary malignancies.

AUTO3 is a bicistronic CD19/22-CAR T previously tested in a phase 1 study in patients with R/R LBCL (ALEXANDER, NCT3289455) (79). The CD19 CAR has a OX40 costimulatory domain, and CD22 has a 4-1BB costimulatory domain (80). Pembrolizumab was used in combination, because PD-L1 upregulation was a possible resistance mechanism demonstrated by previous research. In the study, 52 patients received AUTO3 infusion and 48/52 received pembrolizumab (92.3%); treatment was administered as outpatient in 20 (38.5%) patients. None had previous exposure to CD19- or CD22-targeted therapies. In 47 evaluable patients, ORR was 66.0% and CR 48.9%. However, long-term outcome was not improved by neither AUTO3 nor pembrolizumab due to high rates of relapse, leading to a median PFS was 3.32 months. Response was not associated with AUTO3 expansion. Low serum cytokine levels were observed across the study cohort, correlating with low burden of CRS and ICANS. Previous study of AUTO3 in pediatric B-ALL also demonstrated disappointing results, indicating the necessity for CAR-T optimization (80). Immune checkpoint inhibitors had disappointing results in the treatment of R/R LBCL, which was reaffirmed by the ALEXANDER trial (81).

Tandem CD19/22-CAR T, with both anti-CD19 and anti-CD22 scFv on the extracellular domain, transduced by a single lentiviral vector, are also developed. Among all extracellular designs, tandem CD19/22-CAR with alternative sequence of scFv heavy and light chains, resulting in a loop structure of the extracellular domain, was the most potent one preclinically (82, 83). The loop CAR T subsequently underwent a phase 1 trial, 17 patients with R/R B-ALL and 21 with R/R LBCL received cell infusion. Rate of CRS was 76% (grade≥3, 5%), rate of ICANS was 37% (grade≥3, 10%) and two had laboratory evidence of HLH; all CRS and ICANS resolved. ORR in the LBCL cohort was 62%, half (29%) achieved a CR. Again, the response was short-lived, with median PFS 3.2 months (95%CI, 1.2-5.5). Main cause of resistance/relapse remained antigen loss, but authors also observed that the cells had suboptimal cytokine production against CD22, contributing to CD19-/CD22+ relapse (83). Alternative CD22 scFv and CAR design could potentially improve the efficacy of tandem CD19/22-CAR T (84, 85). Several small single-center clinical trials from China using different CAR constructs in patients with R/R aggressive B-NHL reported seemingly better results (86, 87). Another way to augment CD19/22-CAR T function is through epigenetic modification. Decitabine may enhance CAR-T function and reverse T-cell exhaustion by demethylating silenced genes, and may reverse antigen loss by upregulating antigen expression on lymphoma cells (88, 89). In a retrospective study, adding decitabine to lymphodepletion chemotherapy could improve response rate, DoR and survival in patients receiving CD19/22-CAR T (89).

### CD20/CD22

CAR-T targeting both CD20 and CD22 is attractive for CD19-low or negative relapses. Off-the-shelf allogeneic CAR-T may avoid major hurdles in autologous CAR-T, such as T-cell exhaustion and length production time. Based on these premises, Aranda-Orgilles, et al., designed UCART20x22, an allogeneic, dual CD20 and CD22-targeting CAR-T (90). The allogenic T-cells underwent transcription activator-like effector nuclease (TALEN) mRNA-mediated *CD52* and *TRAC* gene knockout. *CD52*-knockout was to allow the addition of alemtuzumab into lymphodepletion, to avoid rejection; *TRAC*-knockout prevents graft-versus-host disease (91). UCART20x22 is currently being evaluated in NatHaLi-01 (NCT05607420), a phase 1/2 clinical trial for R/R B-NHL. Preliminary results from three treated patients were reported recently. All received DL1. No ICANS or grade ≥ 3 CRS have been observed. At day 28, two achieved CR and one PR (92). NatHaLi-01 has a target sample size of 80 patients and is estimated to complete by November 2027.

### Other targets

#### BAFF and BAFF receptor

B-cell activating factor (BAFF) is crucial for the survival of mature B-cells (93). BAFF has three receptors – BAFFR, TACI, and BCMA – with varying but ubiquitous expression on a variety of B-cell NHLs (94). Due to the importance of BAFF to B-cell survival, antigen escape to BAFF or BAFFR is less likely than CD19 (95). CAR Ts designed against BAFF or BAFFR have both been constructed (95, 96). BAFFR-CAR T demonstrated effective antitumor effect in CD19-negative B-NHL and B-ALL in both *in vitro* cell line and *in vivo* xenograft studies (95). Compared with BAFFR-CAR T, BAFF-CAR T may minimize antigen escape more effectively with the ability to bind to three different receptors (96). Currently, BAFFR-CAR T is being tested in B-ALL or LBL (NCT04690595) while BAFF-CAR T is being tested in B-NHL (NCT05312801).

#### CLL-specific targets - CD23, FCµR, Siglec-6

The treatment paradigm of CLL has shifted dramatically since the development of targeted therapies. However, patients with risk factors such as TP53 mutation, who are refractory to BTKi or BCL2 inhibitors, or those with Richter’s transformation (RT) still have a dismal prognosis. Two issues have hindered the development of CAR-T in CLL. One is the low efficacy of CD19-CAR T in CLL compared with other B-cell malignancies. Different studies reported a CR rate of 20-70% and 18-month PFS of 25% (97). It is suspected that autologous CAR-T suffers from intrinsic T-cell exhaustion, but similar poor response has also been observed in allogeneic CAR-T (98, 99). Other factors may contribute to CLL’s resistance to CAR-T therapy, such as high immune checkpoint protein expression, immune suppressive tumor microenvironment (TME), and high level of circulating inhibitory extracellular vesicles (100). The efficacy data of CD19-CAR T in RT remain scarce and conflicting (101). Some patients may even develop RT after CD19-CAR T, indicating the presence of intrinsic resistance mechanisms in RT to CD19-targeted therapy (102). The other issue with CAR-T development is the risk of B-cell aplasia and prolonged immunosuppression from the “on-target, off-tumor” effect of pan-B-cell targeting. Alternative targets to CD19 have been explored to spare the normal B-cell compartment. CD23, IgM Fc receptor (FCµR, other names include TOSO and Fas-inhibitory molecule 3), and Siglec-6 are proposed targets for CLL. CD23 is typically overexpressed in CLL but not in normal B-cells (103). Preclinical studies demonstrated the efficacy of CD23-CAR T in CLL, which could be enhanced by lenalidomide (104). Similar to CD23, FCµR is highly and consistently expressed by CLL cells but only marginally expressed by normal B-cells and hematopoietic stem cells (HSC) (105). In preclinical models, FCµR-CAR T could eliminate CLL cells while maintaining the number of healthy B-cells (105). Siglec-6 is a CLL surface antigen that is highly restricted in other tissues (106). Anti-Siglec-6 antibody JML-1 has the strongest CLL surface reactivity among detected antibodies in patients with CLL cured by allogeneic hematopoietic stem cell transplant (alloHSCT) (107). Kovalovsky, et al., constructed Siglec-6-CAR T using JML-1-derived scFv, and showed selective cytotoxicity against CLL cells in *in vitro* and *in vivo* xenograft murine models (106). The efficacy and safety of CLL-specific CAR-T need further clarification by clinical trials.

#### CD32B

CD32B is the predominant Fc receptor on B-cells and is expressed on a variety of B-cell malignancies (108). CD32B contributes to resistance development to targeted antibodies such as rituximab, by accelerating internalization of the antibodies (109). Therefore, CD32B is an attractive target for B-cell lymphoma not only from the abundance and specificity, but also for the potential reversal of CD20-resistance. In preclinical models, CAR-T targeting CD32B had effective cytotoxicity against CLL (110). Unlike CD23-CAR T, CD32B-CAR T may not avoid B-cell aplasia because CD32B expression is not limited to CLL. Of note, a small percentage of CD4+ or CD8+ T-cells also express CD32B, which is a T-cell activation suppressor (111). The implication of CD32B expression by T-cells on CD32B-CAR T is unclear.

#### CD70

The CD70-CD27 axis is involved in immune evasion and tumor progression. Aberrant co-expression of CD70 and CD27 has been observed in various B and T-NHL, and high CD70 expression but absence of CD27 has been seen in HL (112). Normal hematopoietic stem cells and most blood cells do not express CD70, making it a desirable CAR target (113). Meanwhile, CD70 contributes to T-cell exhaustion, therefore CD70-CAR T with CD70-knockout may have better performance than CD70-wildtype (114). CD70 is one of the targets of the aforementioned 4SCAR2.0 platform being studied in China (NCT05436496). Eight patients with heavily pretreated DLBCL have been treated with dual CD19 and CD70 targeting CAR T, with CR in 6 and ORR in 7 patients, and median PFS 10.5 months (115). One patient with R/R PCNSL enjoyed ongoing complete remission at 17-month post-4SCART19/70 (116). CD70 is also a viable CAR-NK target but requires CD70-knockout to prevent fratricide (117).

#### CD72

CD72 is a B-cell restricted, highly expressed surface antigen, and is upregulated in B-cell ALL and NHL. Down regulation or loss of CD19 do not impair CD72 expression (118). Different CD72-CAR Ts are undergoing preclinical testing in B-ALL and B-NHL models, showing promising efficacy and no off-target effect (119, 120).

#### CD79

CD79 is a B-cell restricted cell surface heterodimer (CD79a and CD79b, also known as Igα and Igβ) that participate in the B-cell receptor signaling pathway (121). CD79-targeting is an effective strategy in the treatment of B-NHL, exemplified by an anti-CD79b ADC polatuzumab-vedotin. CARs targeting either CD79a or CD79b are under development. JV213 is a CD79b-CAR T with a novel CD79 monoclonal antibody, a CD8α hinge/transmembrane domain, and an OX40 costimulatory domain. In preclinical testing, JV213 was superior to other CD79b-CAR Ts, and a phase 1 clinical trial using JV213 in R/R B-cell lymphomas was initiated (NCT05773040) (122). In a different group, Jiang, et al., introduced an inducible caspase-9 (iCas9) suicidal gene system into CD79b-CAR T to improve safety targets the B-cell receptor component Igβ to avoid antigen escape (123). The iCas9 CD79b-CAR T is undergoing early phase clinical trial in China (NCT05312476). bbT369 is a CD20 and CD79a dual-targeting CAR T which is current being evaluated a multicenter phase 1/2 clinical trial for R/R B-NHL (CRC-403, NCT05169489). In preclinical testing, bbT369 was more potent than CD19-CAR T and induced longer remission (124). Dual-targeting CAR T against CD19 and either CD79a or CD79b CARs are developed (125, 126). Leung, et al., demonstrated that CD19/79a(b)-CAR T induced longer tumor control than single-antigen targeting CAR T from preventing antigen-loss relapse, and targeting CD79a was more potent than C79b. However, bispecific CAR T with either tandem or bicistronic CAR structures had reduced activity against single-antigen positive cells due to compromised antigen binding and signaling, indicating the need to optimize structural design (126). Low-level aberrant CD79b expression monocytes, hematopoietic progenitor cells and T-cells could theoretically cause untoward hematologic toxicity, which will be elucidated by clinical trials (127).

#### ROR1

Receptor tyrosine kinase-like orphan receptor 1 (ROR1) is a cell surface protein overexpressed on various solid and hematological malignancies and minimally expressed on most adult tissues, contributing to cancer stemness (128). To minimize toxicity on hematopoietic stem cells, different switchable CAR-ROR1 have been developed to allow tumor-restricted killing (129, 130). Early phase clinical trials are ongoing to study the efficacy and safety of ROR1-CAR T (ONCT-808) in patients with R/R BCL and/or advanced solid tumors, including those with RT (NCT05694364, NCT05588440) (130, 131). PRGN-3007 is a novel ROR1-CAR T that also includes membrane-bound IL15 for *in vivo* expansion and persistence, a kill switch for safety, and intrinsic PD-1 blockade to enhance cytotoxicity (132). PRGN-3007 is currently undergoing a phase 1/1b clinical trial for both R/R B-NHL and breast adenocarcinomas (NCT0569434) (130).

#### Immunoglobulin light chain CAR

Immunoglobulin is a part of the BCR and participates in BCR signaling upon biding to antigen. Normal B-cells have polyclonal surface immunoglobulin but malignant B-cells are monoclonal. Immunoglobulin light chain κ or λ-targeted CAR takes advantage of the light chain restriction of mature B-cell malignancies, and preserves humoral immunity by sparing normal B-cells with the reciprocal light chain (133). Circulating light chain could help improve CAR-T persistence (134). Dotti, et al, are conducting a phase 1 trial (NCT00881920) studying the safety of κ-CAR T in patients with R/R B-NHL/CLL or MM. Preliminary results on 16 treated patients (B-NHL/CLL = 9, MM = 7) demonstrated that κ-CAR T was well-tolerated without attributable toxicity. CR was achieved in two and PR in one in the B-NHL/CLL cohort (135). The group also constructed λ-CAR T, which in preclinical tests demonstrated light-chain restricted cytotoxicity (136).

#### B-cell maturation antigen

B-cell maturation antigen (BCMA) is not only expressed on multiple myeloma but also on a subset of B-NHL and CLL (137). The manufacture of bispecific CD19/BCMA CAR-T and CAR-NK are both feasible (138, 139). Early phase clinical trial demonstrated the efficacy of CD19/BCMA-CAR-T in multiple myeloma (138). The safety and utility of CD19/BCMA-CAR-T in aggressive B-NHL will be studied in a phase 1 clinical trial (NCT06097455).

#### Tri-specific CAR

CAR-T targeting CD19, CD20, and CD22 could potentially prevent relapse due to antigen loss or down-regulation more effectively. Zhou, et al., designed a tri-specific tandem CAR T that showed stronger cytolytic activity than mono- or bispecific CAR T in preclinical models (140). Schneider, et al., designed a tri-specific CAR T that contained a tandem CD20-CD19 CAR and a second CD22 CAR (141). Costimulatory domain derived from ICOS and OX40 or CD27 was more effective than CD28 or 4-1BB in tri-specific CAR, indicating the importance of optimizing costimulatory domains based on different single-chain variable fragment of the CAR. The tri-specific CAR T with OX40 costimulatory domain has been chosen for a phase 1 clinical trial in patients with R/R B-cell malignancies including NHL and CLL (NCT05418088) (142).

## Classical Hodgkin lymphoma

Classical Hodgkin lymphoma (cHL) is characterized by a small percentage of malignant Hodgkin and Reed-Sternberg (HRS) cells that are of mature B-cell origin, embedded within a highly immunosuppressive, HRS-induced TME (143). Despite recent advances, patients with R/R cHL, especially those with prior exposure to CD30-ADC brentuximab vedotin and checkpoint inhibitors, still have a dismal prognosis. CAR-based therapy is a potential therapeutic option for these patients. The development of CAR in HL has been focused on targeting surface antigens of HRS cells and/or reversal of immune evasion (144). Active clinical trials of novel CAR-based therapy in cHL are listed in **Table 2**.

**Table 2:** Active clinical trials of novel CAR therapy for Hodgkin Lymphoma and T-cell lymphoma.

CAR Target	NCT number	Disease	Phase	Location	Notes
CD4	NCT03829540	CD4-positive ALL and NHL	1	US - IU and Stony Brook Cancer Center	
CD5	NCT04767308	CD5-positive NHL	1	China	
	NCT03081910	CD5-positive T-cell leukemia and lymphoma	1	US - Baylor	Two arms: AutoCAR and AlloCAR; AlloCAR is from prior allogeneic transplant donor
	NCT05138458	CD5-positve T-cell lymphoma	1 and 2	US - multicenter	
CD7	NCT04599556	CD7-positive hematologic malignancy	1 and 2	China	
	NCT04840875	CD7 positive ATLL and T-ALL	1	China	
	NCT04823091	CD7-positive T-cell leukemia and lymphoma	1	China	AlloCAR
	NCT04689659	CD7-positive T-cell leukemia and lymphoma	2	China	AlloCAR
	NCT05059912	CD7-positive T-cell lymphoma	2	China	
	NCT05377827	CD7-positive malignancies including T-NHL or AML	1	US - Wash U	AlloCAR
	NCT03690011	CD7-positive T-cell lymphoma	1	US - Baylor	
CD30	NCT02259556	CD30-positive HL and NHL	1 and 2	China	
	NCT04653649	HL and CD30-positive ALCL and PTCL	1 and 2	Spain	Memory T-cells enriched, proximal target on CD30
CD30	NCT02917083	CD30-positive malignancy	1	US - Baylor	
	NCT02690545	CD30-positve HL and NHL	1 and 2	US - UNC	Flu/Benda LDC
	NCT06090864	CD30-positive HL	1 and 2	US - UNC	Co-expressing CCR4; Flu/Benda LDC
CD30	NCT03602157	CD30-postive HL and CTCL	1	US - UNC	Co-expressing CCR4; Flu/Benda LDC
	NCT04288726	CD30-positive HL, PTCL, other aggressive NHL	1	US - Baylor	AlloCAR using EBV-specific T-cells
CD37	NCT04136275	CD37-positive HL and NHL	1	US - MG	DLT at doses ≥ 100 million/kg, with bone marrow aplasia requiring allogeneic stem cell rescue
CD147	NCT05013372	CD147-positive T-NHL	1	China	AlloHSCT eligibility and donor availability are required
TRBC1	NCT04828174	TRBC1 positive T-cell malignancy	1	China	Suspended due to inability to enroll qualified patients
	NCT03590574	TRBC1 positive T-NHL	1 and 2	Europe - multicenter	

US, United States; ALL, acute lymphoblastic leukemia; NHL, non-Hodgkin Lymphoma; IU, Indiana University; AutoCAR, autologous CAR-T; AlloCAR, allogeneic CAR-T; Wash U, Washington University in St. Louis; HL, Hodgkin lymphoma; ALCL, anaplastic large cell lymphoma; PTCL, peripheral T-cell lymphoma; UNC, University of North Carolina; MG, Massachusetts General Hospital Cancer Center; DLT, dose-limiting toxicity; alloHSCT, allogeneic hematopoietic stem cell transplant.

### CD30

CD30 is one of the most extensively studied targets on cHL due to its strong and restricted expression on HRS. CD30 expression is upregulated on activated B- and T-cells (145). CD30 plays an anti-apoptotic and immunosuppressive role in lymphoma and TME, and may trigger chromosome instability and mutations in lymphoma cells (146). The study of CD30-CAR T in cHL began in the late 1990s, but the clinical application has been plagued by suboptimal response rate and duration of response, albeit generally good tolerance Based on pre-clinical data of a CD30-CAR T construct using a mouse-derived anti-CD30 monoclonal antibody as scFv, Ramos, et al., conducted a phase 1 clinical trial enrolling nine patients with heavily pretreated Epstein-Barr virus (EBV)-, CD30+ lymphoma, including seven with cHL (NCT01316146) (147, 148). Lymphodepletion chemotherapy (LDC) was not given. CD30-CAR T was well tolerated. Two patients with cHL had durable CR, the rest had transient SD. Subsequently, two parallel phase 1/2 studies of CD30-CAR T in patients with R/R CD30+ lymphoma were conducted, enrolling 41 adult patients with heavily pretreated cHL (NCT02690545, NCT02917083) (149). LDC regimens included bendamustine ± fludarabine, and fludarabine/cyclophosphamide. Among evaluable patients, ORR was 72% and CR 59%, 1-year PFS was 41%. Cutaneous and hematological toxicities were notable after CD30-CAR T infusion. No ICANS was seen and all CRS cases were low grade. Bendamustine/fludarabine LDC conferred the lowest rate of CRS and best response, and was selected for an ongoing multicenter phase 2 CHARIOT study evaluating CD30-CAR T in R/R cHL who failed at least 3 prior lines of therapy including chemotherapy, brentuximab-vedotin and PD-1 inhibitor (NCT02259556) (150).

Wang, et al., conducted a phase 1 clinical trial in China using a CD30-CAR T with different anti-CD30 scFv (NCT02259556) for patients with R/R CD30+ lymphoma (151). Eighteen patients were enrolled, including 17 with cHL and 1 with primary cutaneous anaplastic large-cell lymphoma (ALCL). Therapy was well tolerated however none of the patients achieved CR. PR rate was 39% and median PFS was 6 months (range, 3-14 months). Extranodal disease had poor response to CD30-CAR T. Another phase 1 study in China utilized a CD30-CAR T with dual CD28 and 4-1BB costimulatory domains and post-CAR anti-PD-1 consolidation (152). Among 6 patients with R/R cHL, 1 died due from early post-CAR-T pleural hemorrhage, 5 achieved CR, but only 1 enjoyed ongoing remission beyond 3 years, the rest relapsed within 10 weeks to 28 months. Although the sample size was small, it appeared that dual costimulatory domain improved CD30-CAR T persistence but did not lead to increased toxicity.

Major barriers to the success of CD30-CAR T include off-target elimination of other CD30-expressing cells such as other T-cells, inefficient homing to tumor, intrinsic resistance of the tumor cells, and CD30-downregulation (153, 154). Whether soluble CD30 affect CAR function remains controversial (147, 148). A Spanish group developed a CD30-CAR T that targets a proximal epitope of CD30 to avoid interaction with soluble CD30 and enriched the product in memory T-cells. Preliminary results showed a 100% overall response rate and 50% durable CR rate in 10 patients who received therapy (155). To improve homing to lymphoma and persistence of CAR-T, strategies include co-expression of CCR4 or dual costimulatory domain of CD28/4-1BB (156, 157). CCR4-expressing CD30-CAR T is being evaluated in a phase 1 clinical trial (NCT03602157) (158). Preliminary data on 8 evaluable patients with cHL demonstrated 100% ORR with CR in 75%. Patient experienced no dose-limiting toxicity and the duration of response was prolonged – at a median follow up of 12.7 months, the median PFS was not reached and one CR was ongoing beyond 2.5 years. To reverse intrinsic immune suppression in HL, post-CD30-CAR T PD-1 blockade has been studied in multiple studies showing improved expansion of CD30-CAR T and duration of response (152, 159, 160). Autologous stem cell transplant may have synergistical effects with CD30-CAR T and could consolidate and prolong the remission post-CD30-CAR T (161).

As previously tested CD30 scFv were derived from murine antibodies, there is a concern of human anti-mouse antibody causing resistance to CD30-CAR T. Fully-humanized CD30-CAR T was developed and tested in a phase 1 clinical trial at the NIH but with disappointing results (162). Among 21 patients treated, 20 had cHL; ORR was 43% and CR only occurred in 1 patient. Median duration of response was 8.9 weeks. The study was stopped early due to prolonged cutaneous and hematological toxicities. It was speculated that high disease burden and poor penetration of CAR-T to lymphoma due to immunosuppressive microenvironment were the main reasons for low efficacy.

### Other targets

Several cell surface markers in the cHL TME have been proposed as CAR targets to break the immunosuppressive cycle. CD19+ B-cells are part of the cHL TME and some CD19+ B-cells may be HRS stem cells, making CD19 a putative target for CAR-T in cHL (163). Svoboda, et al., conducted a pilot study using a nonviral messenger RNA-vector transduced CD19-CAR T in patients with R/R cHL (NCT02277522 and NCT02624258) (163). Nonviral vector transduced CAR was only expressed for a few days as opposed to the persistent expression of viral-transduced CAR, potentially reducing the toxicity. The treatment was well tolerated but response was limited and short-lived. Of 4 treated patients, only 1 achieved CR but relapsed within 3 months. cHL positive for both CD19 and CD30 account for about 5% of all cases, making lentiviral-transduced sequential CD19-CAR T and CD30-CAR T is another therapeutic strategy (164). In a case report, one patient with CD19+CD30+ cHL had PR with CD19-CAR T and further response after subsequent CD30-CAR T (164). CD20 expression is more common in cHL, comprising around 20% of all cases and the prevalence is higher in EBV+ cHL (165, 166). Anti-CD20 therapy with rituximab is effective in CD20+ cHL (167). it is plausible that CD20 can become a CAR target for patients with CD20+ cHL.

CD123 is the α-subunit of interleukin-3 receptor and is widely expressed both on HRS and on the tumor-associated macrophages in the TME (168). In a preclinical study by Ruella, et al., CD123-CAR T may target HRS directly and overcome the immunosuppressive TME, killing cHL both *in vitro* and *in vivo*, and establish long-term memory (169). Due to expression of CD123 on normal hematopoietic cells and endothelial cells, off-target toxicity such as bone marrow failure is a valid concern for clinical application.

## T-cell lymphoma

Development of CAR T-cell therapy in T-cell malignancies have been limited by fratricide, i.e., killing of sibling CAR and normal T-cells, leading to reduced efficacy and profound immune suppression (170). Identification of malignancy-specific T-cell surface antigen, such as C-C motif chemokine receptor 9, may circumvent fratricide (171). Some antigens are shared between B and T-cell lymphomas, such as CD37 and CD38, and no significant fratricide has been seen with CAR-T targeting CD37 or CD38 in preclinical experiments (172). Another hurdle in CAR T development is the risk of contamination by malignant T-cells in autologous products (173). Further, functional T-cells may be absent in patients with advanced T-NHL, leading to poor autologous T-cell quality (174). AlloCAR, on the other hand, may introduce GvHD. T-cell receptor alpha constant (TRAC)-knockout can prevent GvHD and is often employed in the construction of alloCAR (175). Tyrosine kinase inhibitors dasatinib and ibrutinib may suppress fratricide, enhance anti-tumor activity, and promote expansion (176, 177). Another promising development is CAR-NK which are non-GvHD inducing, off-the-shelf, and can be modified to prevent fratricide (174, 178). Active clinical trials of novel CAR-based therapy in TCL are listed in **Table 2**.

### CD5

CD5 is present on at least 80% of T-cell malignancies, thymocytes, peripheral T-cells, and a small proportion of B-cells. Mamonkin, et al., constructed CD5-CAR T with CD28 costimulatory domain had attractive features in preclinical experiments, namely limited and transient fratricide and preserved immune response to viral antigens, but the cells failed to eliminate malignant T-cells *in vivo* and animals developed CD5+ relapse (179). CD5-CAR T with 4-1BB costimulatory domain exhibited better antitumor activity but with increased fratricide. To overcome fratricide, Mamonkin et al., designed reversible CAR expression system that could be suppressed with small molecules during *in vitro* cell culture, and restore CAR expression *in vivo* after drug withdrawal (180). Based on promising pre-clinical studies, Hill and Mamonkin, et al., conducted a phase 1 clinical trial applying autologous CD5-CAR T with CD28 costimulatory domain to patients with R/R T-cell malignancies (NCT0308190) (181). Among 17 enrolled patients with heavily pretreated TCL, 2 died before cell manufacture and 2 died prior to infusion, 2 received alternative therapy, I failed eligibility, and 1 production failed, rendering a total of 9 patients who received cell infusion. Therapy was well tolerated, with toxicity profile similar to commercial CD19-CAR T. Response was observed in 4 patients, including two CRs that lasted 6.4 and 7.2 months in the absence of consolidative alloHSCT (181). The study highlighted the challenges in timely manufacture of CAR-T in this heavily pre-treated population. Off-the-shelf alloCAR and CAR-NK may be more readily available for patients. Donor-derived CD5-knockout CD5-CAR T has been evaluated in a phase 1 clinical trial in patients with R/R T-ALL (182). All patients were previously treated with CART7 and had CD7-negative relapse. MRD-negative CR was achieved in all patients but the follow up was limited. Most of the side effects were hematological, but 1 patient developed lethal EBV infection with HLH at 2.7 months post therapy. NK-92 cells transduced with CD5-CAR have demonstrated effective antitumor activity in murine T-ALL/TCL xenograft models (183, 184).

### CD7

Autologous and allogeneic CD7-CAR Ts (autoCD7 and alloCD7, respectively) have both been developed. Most of the CD7-CAR T clinical trials have focused on T-acute lymphoblastic leukemia (ALL)/lymphoblastic lymphoma. An off-the-shelf alloCD7 product, WU-CART-007, utilized CRISPR/Cas9 deletion of CD7 and TRAC to minimize fratricide and GvHD (185). WU-CART-007 is undergoing a global phase 1/2 clinical trial in T-ALL/LBL (NCT04984356) (186). Pan, et al., conducted a phase 1 clinical trial in China focusing on T-ALL/LBL with post-alloHSCT relapse (187). T-cells were harvested from original donor (n = 12) or new donor (n = 8). The lentiviral vector had a CD7 binding domain to retain CD7 intracellularly and prevent fratricide. CRS and GvHD were common but mostly low grade. ICANS occurred in 15%, all < grade 3. All patients had grade 3-4 cytopenia likely related to the nature of disease. CR was seen in 90% of patients and 83% of the CR were durable at a median follow up of 6.3 months. New donor-derived CD7-CAR T did not cause higher rate of GvHD due to mixed chimerism. The authors also observed T-cell immune reconstitution from CD7-negative T-cells.

Another phase 1 study from China compared the outcomes of patients with T-cell malignancies, including 8 T-ALL/LBL and 2 PTCL, who received autoCD7 (n = 5) or alloCD7 (n = 5)(NCT04823091) (188). Notable toxicities included CRS in 8 patients, grade 3 CRS in 1, and HLH in 2. No ICANS was observed. Two patients experienced mild GvHD. Majority of patients had significant pancytopenia and/or infection complications. Unfortunately the two patients with PTCL did not respond. AlloCD7 was more readily available and did not require washout between chemotherapy and leukapheresis, benefiting patients with rapidly progressing refractory disease. Compared with autoCD7, alloCD7 was associated with higher response rate, less relapse, and better CAR T persistence (188). For patients with T-ALL/LBL Allogeneic stem cell transplantation post-alloCD7 was shown to be safe, and patients with CD7-positive relapse post-transplant could achieve remission again with alloCD7 re-treatment (186, 189).

Fratricide resistant CD7-CAR T may also be produced through natural selection of minimal CD7 epitope expressing T-cells from bulk T-cells either *in vitro* or *in vivo* (177, 190). Dasatinib and ibrutinib can temporarily inhibit fratricide and facilitate *ex vivo* expansion (177). CD7 is a viable target for CAR-NK and different NK-92-based CD7-CAR NKs have been developed in the lab pending clinical verification (191, 192).

Relapse after CD7-CAR T is usually from antigen escape which may be prevented by dual CD5/CD7 targeting (187, 193). In a preclinical study, Dai, et al., transduced CD5, CD7 or tandem CD5/CD7 scFv to CD5/CD7 knockout T-cells. CD5 and CD7 knockout did not change TCR structure and prevented fratricide. Tandem CD5/CD7 CAR-T had the best *in vivo* antitumor activity and prolonged the survival of mice bearing xenograft in murine xenograft models (193).

### CD2

Costimulatory receptor CD2 is commonly expressed on T- and NK-cell surface and is involved in T-cell development and function (194). CD2 is expressed in about 90% adult peripheral T-cell lymphoma and about 70% in pediatric T-acute lymphoblastic leukemia/lymphoma (ALL/LBL) and PTCL, higher than CD7 (40-50%) (195). The development of CAR2 is potentially limited by fratricide, necessitating CD2-knockout in CAR-T. Recently, Angelos et al., tested CD2-knockout autologous CD2-CAR T in preclinical models, showing high antitumor activity even in post-CAR5 relapsed T-ALL xenograft mice (195). Xiang et al., developed a CD2 and TCR alpha subunit knockout allogeneic CAR T against CD2 (UCART2) to minimize fratricide and GvHD. CD2-knockout led to reduced CAR-T function, which could be overcome by coadministration of IL-7. Preclinical study demonstrated that the combination of UCART2 and IL-7 could effectively prolong the survival of xenograft mouse model with T-cell malignancy (196).

### CD147

CD147 is highly expressed in several types of solid tumor and T-cell malignancies. Aside from promoting invasion and metastasis, CD147 is indispensable for T-cell differentiation at the thymus level (197). Several CD147-CAR Ts of various designs are undergoing different stages of clinical development in hepatocellular carcinoma and non-small cell lung cancer (198–200). CD147-CAR T exhibited potent efficacy and absent off-target effect in cell-line and xenograft models (201). An early phase clinical trial (NCT05013372) is being conducted in China to evaluate the safety and efficacy of CD147-CAR T in CD147-positive R/R T-NHL.

### T-cell receptor-based therapy

T cell receptor β-chain constant domains 1 and 2 (TRBC1 and TRBC2) expression are mutually exclusive on T-cell surface. TRBC1 is expressed by around 40% of normal T-cells, and the incidence of TRBC1 positivity in T-cell malignancies is similar (202, 203). TRBC-targeting can eliminate the cancer and normal T-cells that express the specific TRBC but spare the other group of normal T-cells, thereby limiting fratricide, and rescuing patients from intolerable immunosuppression (204). An ongoing phase 1/2 clinical trial (NCT03590574) demonstrated that autologous TRBC1-CAR T was well-tolerated, and at a higher dose could induce response in patients with R/R TRBC1-positive PTCL (203). Initially, duration of response was short, with a lack of circulating CAR T expansion. The manufacturing process was modified to produce a more naïve phenotype, improving the duration of response (205). Another method that may improve TRBC1-CAR T function is to only transduce pre-selecting TRBC1-negative T-cells, to prevent fratricide and contamination of the product by TRBC1-positive malignant cells (202). Updated results from clinical trials are eagerly anticipated to further elucidate the utility of autologous TRBC1-CAR T.

TCR receptor variable region have also been proposed as potential CAR targets. T-cell malignancy typically exhibit TCR variable β-chain (Vβ) clonality, therefore targeting malignancy-specific Vβ may achieve cancer-specific cytotoxicity while sparing other normal T-cells. Vβ-targeting CAR-T and CAR-NK have both been generated, with proof-of-concept preclinical results (174, 206). TCR mutation or down-regulation may confer resistance to TCR-targeting therapy. Some T-NHLs do not have αβ TCR but have γδ TCR, and those would not respond to therapy against TRBC or Vβ.

### Other targets

Pan-T antigen CD3 is a target of interest in T-cell malignancies but antibody-based therapies have been unsuccessful (207). Gene-edited T-cells with disrupted CD3 expression can be made into CD3 CAR-T that are resistant to fratricide. CD3 is also an appealing target for CAR-NK as NK-cells do not express CD3. Several pre-clinical studies have demonstrated strong antitumor activity of CD3-targeted, CD3-knockout CAR-T or CAR-NK in preclinical studies (208–210).

CD4 is a ubiquitous marker of mature T-cells. CD4-targeting CAR T with an alemtuzumab safety switch is currently in clinical trial for CD4-positive R/R T-cell NHL and ALL. Preliminary results demonstrated the efficacy and safety of CD4-CAR T (211). CD4 is also a potential CAR-NK target (212).

CD30 is ubiquitously expressed in systemic ALCL and variably expressed in other PTCL (213). Although CD30-CAR T is mainly studied in cHL, several patients with ALCL were enrolled in different trials, with good tolerance, minimal fratricide, and some patients enjoyed durable CR (147, 151, 152). Aside from ALCL, Voorhees, et al., reported durable CR after CD30-CAR T in a patient with heavily pre-treated CD30+ enteropathy-associated T-cell lymphoma (214). Of note, the patient previously received alloHSCT so the CD30-CAR T was of allogeneic origin.

NKG2D-ligand is highly expressed on cancer cells and rarely on healthy tissue. The NKG2D/NKG2D-ligand interaction is important for NK-cell mediated immune surveillance, but contributes to NK-cell exhaustion in cancer (215). T-cells transduced with NKG2D has become an exciting tumor-agnostic treatment for cancer. In NKG2D-ligand deficient tumor, NKG2D-T may also induce tumor-specific immunity by enhancing immune surveillance and modifying TME (216). Preclinical study demonstrated efficacy of NKG2D-T in T-NHL cell lines (216). Currently, several clinical trials are actively testing the efficacy and safety of NKG2D-T in solid tumors and AML/MDS (217).

CCR4 is a chemokine receptor expressed mainly in Tregs. Anti-CCR4 monoclonal antibody mogamulizumab has proven efficacy in T-reg-derived malignancies such as adult T-cell leukemia/lymphoma (ATLL) and CTCL. Perera, et al., demonstrated preclinical *in vitro* and *in vivo* efficacy of CCR4-CAR T in ATLL and other CCR4-positive TCL (218). Contrary to other CAR T, fratricide can improve the quality of CCR4-CAR T by eliminating Treg and type-2 helper T-cells, while sparing cytotoxic CD8+ and type-1 helper T-cells, enhancing antitumor efficacy (219).

## Tumor-agnostic CAR

Certain molecules are expressed in different types of lymphoma and can become tumor-agnostic targets for lymphomas sharing the same marker. Besides CD30, which is shared by both cHL and TCL, other molecules of interest include CD37, CD38, and EBV-associated protein.

CD37 is highly expressed on universally all B-NHL and some cutaneous T-cell lymphoma (CTCL) and peripheral T-cell lymphoma (PTCL), while absent on hematopoietic stem cells, making it a potentially feasible CAR target (172). Clinical trials utilizing anti-CD37 monoclonal or bispecific antibodies and ADC in R/R B-NHL have been disappointing in general, likely due to the close association between CD20 and CD37 (220). In CD20-down regulated B-NHL, CD37 expression is also decreased, impairing the function of antibody-based therapy. Although CAR T typically requires high antigen expression, CD37 down-regulation do not seem to affect the function of CD37-CAR T (220). Preclinical study demonstrated potent cytotoxicity of CD37-CAR T against various CD37-expressing B- and T-NHL, without T-cell fratricide or detectable toxicity to other immune cells such as NK-cells and monocytes (29, 172). Structural modification, such as dual-costimulatory domain and novel linker design, may further improve CD37-CAR T function (221). A phase 1 clinical trial (NCT04136275) treated four heavily pre-treated patients (2 HGBCL, 1 CTCL, 1 HL) with CD37-CAR T, and achieved prolonged CR in three. However, two patients developed bone marrow failure unexpectedly, likely due to high T-cell dose (222). Bispecific CD19/37-CAR Ts were developed by several independent groups, yet to be tested in clinical trials (172, 223, 224).

CD38 signals multiple immunoregulatory pathways and is expressed by various hematological malignancies including MCL, LPL, Burkitt lymphoma, CTCL and NK/T-cell lymphoma (225–228). Lymphoma cells highly expressing CD38 respond well to CD38-CAR T, and those dimly expressing CD38 could be re-sensitized to CD38-targeted therapy by all-trans retinoic acid or panobinostat (228–230). Single targeting CD38-CAR T and dual targeting tandem CD38/latent membrane protein-1-CAR T all showed promising cytotoxicity against NK/T-cell lymphoma in *in vitro* and *in vivo* pre-clinical experiments (178). CD38-CAR NK has also been explored. Due to high CD38 expression on NK-cells, CD38-CAR NK needs simultaneous CD38-knockout to avoid fratricide (229, 231). CD38-CAR NK is effective against various CD38-expressing hematological malignancies in preclinical testing (229, 231).

Ebstein-Barr virus (EBV) is associated with various solid and hematologic malignancies. It is estimated that about 8% of lymphomas are EBV-positive, with the highest rate in angioimmunoblastic T-cell lymphoma, close to 50%, followed by about 30-40% in cHL; in immunocompromised hosts, lymphomas are almost universally positive for EBV (232–234). EBV contributes to pathogenesis and generation of immunosuppressive TME, and can be a marker of relapse (233, 235). Latent membrane proteins (LMP1 and LMP2A) are proteins encoded by EBV and participate in oncogenesis (236). A series of clinical trials were conducted using LMP1/2-specific cytotoxicity T-lymphocytes for cHL, demonstrating an ORR around 30-60% (237). CAR-T targeting LMP showed preclinical efficacy in LMP-positive solid and hematological malignancies, such as ENKL (178, 238, 239). Gp350, an abundant EBV envelop glycoprotein, is another potential target to treat EBV lymphoproliferative disease (240).

Other strategies of tumor-agnostic CAR design pertain to improving cell trafficking to tumor and recognition of tumor antigen. For example, tumoral injection of a substance followed by administration of CAR-T specific to the substance can facilitate CAR-T homing (241). This strategy is more applicable to limited number of solitary lesions especially in solid tumor. Such substance could be small molecules that can be inserted into cell membrane by liposomal vector, or CD19 that can be transduced via oncolytic virus (241, 242). Another strategy is to improve T-cell function, to enhance host immunity against malignancy. Lai, et al., designed a CAR-T that secrets dendritic cell growth factor Fms-like tyrosine kinase 3 ligand (Flt3L) that can recruit host dendritic cells, increase T-cell activation, and induce epitope spreading towards otherwise unexposed tumor antigens (243).

CD16-expressing T-cells replaces the directly antigen-recognizing scFv with the extracellular portion of the Fc gamma receptor CD16, adding NK-cell like function to T-cells. When co-administered with tumor-specific antibody, the CD16-T recognizes the opsonized tumor cells and exerts cytotoxicity via antibody-mediated cellular cytotoxicity (244). When toxicity occurs, treatment can be aborted by withdrawal of antibody (245). While the presence of tumor antigen and respective monoclonal antibody are still required, CD16-T may overcome resistance mechanisms such as NK-cell exhaustion or lack of NK-cell infiltration in the TME (246). ATTCK-20-03 (NCT03189836) is a phase 1 clinical trial evaluated the combination of CD16-T and rituximab for R/R B-NHL (247). Among the 25 subjects treated, 14 (56%) responded, including 10 (40%) CR, with the longest duration of CR 586 days (range, 85-586). Treatment was well tolerated, resulting in no DLT, 1 case of grade 3 neutropenia, and 1 case of grade 2 CRS. ATTCK-20-03 demonstrated that antibody/CD16-T coupling is a feasible approach towards cancer treatment.

## Conclusion

Identification of novel tumor antigens opens new therapeutic avenues for B-cell NHL beyond approved CD19-CAR T, and extends the application of CAR-based therapy to HL and TCL. Dual or multi-targeted CAR may lower the risk of antigen escape-mediated relapse. Tumor agnostic CAR may broaden the indication of adoptive cellular therapy across tumors of different cellular origin. Yet, finding new targets is not the only way to improve the feasibility and efficacy of CAR-based therapy. Modification of the non-antigen biding domains on a CAR may improve cell persistence and reduce toxicity (248). Genetic modification outside of the CAR molecule, such as adding Toll-like receptor, IL-18 expression, adding IL-7 and CCL19 expression, or administration of cytokines *in vivo*, may improve the activity of cells and prolong persistence (249–252). Vaccination combined with viral-specific CAR-T, immune checkpoint modification, pre-selection of memory- and naïve T-cells and elimination of Tregs, may reduce T-cell exhaustion and immunosuppression (63, 253–256). Advanced cell engineering enables the incorporation of inducible CAR expression switches to reduce toxicity (257). New manufacturing platforms reduce the cost and vein-to-vein time, improving the accessibility of CAR-T (258, 259). The rapidly evolving field of CAR-based therapy should hopefully deliver products with better efficacy, tolerability and accessibility, broader indication, and less physical and financial toxicity to patients with lymphoma.

## Author contributions

YP: Writing – original draft, Writing – review & editing. NG: Writing – review & editing.

## References

[1] Labanieh L Mackall CL. CAR immune cells: design principles, resistance and the next generation. Nature. (2023) 614:635–48. doi: 10.1038/s41586-023-05707-3 36813894

[2] Sadelain M Brentjens R Rivière I. The basic principles of chimeric antigen receptor design. Cancer Discovery. (2013) 3:388–98. doi: 10.1158/2159-8290.CD-12-0548 PMC3667586 23550147

[3] Watanabe K Terakura S Martens AC van Meerten T Uchiyama S Imai M. Target antigen density governs the efficacy of anti–CD20-CD28-CD3 ζ Chimeric antigen receptor–modified effector CD8+ T cells. J Immunol. (2015) 194:911–20. doi: 10.4049/jimmunol.1402346 25520398

[4] Cappell KM Kochenderfer JN. Long-term outcomes following CAR T cell therapy: what we know so far. Nat Rev Clin Oncol. (2023) 20:359–71. doi: 10.1038/s41571-023-00754-1 PMC10100620 37055515

[5] Fischer JW Bhattarai N. CAR-T cell therapy: mechanism, management, and mitigation of inflammatory toxicities. Front Immunol. (2021) 12:693016. doi: 10.3389/fimmu.2021.693016 34220853 PMC8250150

[6] Michelozzi IM Gomez-Castaneda E Pohle RVC Cardoso Rodriguez F Sufi J Puigdevall Costa P. Activation priming and cytokine polyfunctionality modulate the enhanced functionality of low-affinity CD19 CAR T cells. Blood Advances. (2023) 7:1725–38. doi: 10.1182/bloodadvances.2022008490 PMC10182295 36453632

[7] Kath J Du W Pruene A Braun T Thommandru B Turk R. Pharmacological interventions enhance virus-free generation of TRAC-replaced CAR T cells. Mol Ther Methods Clin Dev. (2022) 25:311–30. doi: 10.1016/j.omtm.2022.03.018 PMC9062427 35573047

[8] Duell J Leipold AM Appenzeller S Fuhr V Rauert-Wunderlich H Da Via M. Sequential antigen loss and branching evolution in lymphoma after CD19- and CD20-targeted T-cell–redirecting therapy. Blood. (2024) 143:685–96. doi: 10.1182/blood.2023021672 PMC10900140 37976456

[9] Neelapu SS Rossi JM Jacobson CA Locke FL Miklos DB Reagan PM. CD19-loss with preservation of other B cell lineage features in patients with large B cell lymphoma who relapsed post-axi-cel. Blood. (2019) 134:203. doi: 10.1182/blood-2019-126218

[10] Plaks V Rossi JM Chou J Wang L Poddar S Han G. CD19 target evasion as a mechanism of relapse in large B-cell lymphoma treated with axicabtagene ciloleucel. Blood. (2021) 138:1081–5. doi: 10.1182/blood.2021010930 PMC8462361 34041526

[11] Cheng Q Tan J Liu R Kang L Zhang Y Wang E. CD20-specific chimeric antigen receptor-expressing T cells as salvage therapy in rituximab-refractory/relapsed B-cell non-Hodgkin lymphoma. Cytotherapy. (2022) 24:1026–34. doi: 10.1016/j.jcyt.2022.05.001 35691818

[12] Smith MR. Rituximab (monoclonal anti-CD20 antibody): mechanisms of action and resistance. Oncogene. (2003) 22:7359–68. doi: 10.1038/sj.onc.1206939 14576843

[13] Johnson NA Leach S Woolcock B deLeeuw RJ Bashashati A Sehn LH. CD20 mutations involving the rituximab epitope are rare in diffuse large B-cell lymphomas and are not a significant cause of R-CHOP failure. Haematologica. (2009) 94:423–7. doi: 10.3324/haematol.2008.001024 PMC2649361 19211644

[14] Sar A Perizzolo M Stewart D Mansoor A DiFrancesco LM Demetrick DJ. Mutation or polymorphism of the CD20 gene is not associated with the response to R-CHOP in diffuse large B cell lymphoma patients. Leuk Res. (2009) 33:792–7. doi: 10.1016/j.leukres.2008.10.013 19054557

[15] Budde LE Olszewski AJ Assouline S Lossos IS Diefenbach C Kamdar M. Mosunetuzumab with polatuzumab vedotin in relapsed or refractory aggressive large B cell lymphoma: a phase 1b/2 trial. Nat Med. (2023) 30:229–39. doi: 10.1038/s41591-023-02726-5 PMC10803244 38072960

[16] Till BG Jensen MC Wang J Chen EY Wood BL Greisman HA. Adoptive immunotherapy for indolent non-Hodgkin lymphoma and mantle cell lymphoma using genetically modified autologous CD20-specific T cells. Blood. (2008) 112:2261–71. doi: 10.1182/blood-2007-12-128843 PMC2532803 18509084

[17] Till BG Jensen MC Wang J Qian X Gopal AK Maloney DG. CD20-specific adoptive immunotherapy for lymphoma using a chimeric antigen receptor with both CD28 and 4-1BB domains: pilot clinical trial results. Blood. (2012) 119:3940–50. doi: 10.1182/blood-2011-10-387969 PMC3350361 22308288

[18] Shadman M Gopal AK Smith SD Lynch RC Ujjani CS Turtle CJ. CD20 targeted CAR-T for high-risk B-cell non-hodgkin lymphomas. Blood. (2019) 134:3235. doi: 10.1182/blood-2019-125102

[19] Shadman M Yeung C Redman M Lee SY Lee DH Ra S. Safety and efficacy of third generation CD20 targeted CAR-T (MB-106) for treatment of relapsed/refractory B-NHL and CLL. Blood. (2021) 138:3872. doi: 10.1182/blood-2021-149181

[20] Shadman M Caimi PF O'Brien SM Reagan PM Dezube B Navaratnarajah P. Efficacy and safety of a third generation CD20 CAR-T (MB-106) for treatment of relapsed/refractory indolent B-cell non-hodgkin lymphoma: phase-1 results from a multicenter trial. Blood. (2023) 142:2102. doi: 10.1182/blood-2023-175007

[21] Li P Liu W Ye S Zhou L Zhu J Huang J. Two-year follow-up results of C-CAR066, a novel anti-CD20 chimeric antigen receptor cell therapy (CAR-T) in relapsed or refractory (r/r) large B-cell lymphoma (LBCL) patients after failure of CD19 CAR-T therapy. Blood. (2023) 142:2115. doi: 10.1182/blood-2023-181527

[22] Kutsch N Gödel P Holtick U Lohneis A Vucinic V Altefrohne FP. A phase I dose finding trial of MB-CART20.1 in patients with relapsed or refractory B-cell non-hodgkin lymphoma. Blood. (2022) 140:12980–1. doi: 10.1182/blood-2022-168730

[23] Shah NN Sokol L. Targeting CD22 for the treatment of B-cell Malignancies. Immunotargets Ther. (2021) 10:225–36. doi: 10.2147/ITT.S288546 PMC8275043 34262884

[24] Dhillon S. Moxetumomab pasudotox: first global approval. Drugs. (2018) 78:1763–7. doi: 10.1007/s40265-018-1000-9 PMC6323103 30357593

[25] Kantarjian HM DeAngelo DJ Stelljes M Martinelli G Liedtke M Stock W. Inotuzumab ozogamicin versus standard therapy for acute lymphoblastic leukemia. New Engl J Med. (2016) 375:740–53. doi: 10.1056/NEJMoa1509277 PMC5594743 27292104

[26] Fayad L Offner F Smith MR Verhoef G Johnson P Kaufman JL. Safety and clinical activity of a combination therapy comprising two antibody-based targeting agents for the treatment of non-hodgkin lymphoma: results of a phase I/II study evaluating the immunoconjugate inotuzumab ozogamicin with rituximab. J Clin Oncol. (2013) 31:573–83. doi: 10.1200/JCO.2012.42.7211 PMC4878046 23295790

[27] Dang NH Ogura M Castaigne S Fayad LE Jerkeman M Radford J. Randomized, phase 3 trial of inotuzumab ozogamicin plus rituximab versus chemotherapy plus rituximab for relapsed/refractory aggressive B-cell non-Hodgkin lymphoma. Br J Haematol. (2018) 182:583–6. doi: 10.1111/bjh.14820 PMC6200343 28677896

[28] Echeverri C Fisher S King D Craig FE. Immunophenotypic variability of B-cell non-Hodgkin lymphoma: a retrospective study of cases analyzed by flow cytometry. Am J Clin Pathol. (2002) 117:615–20. doi: 10.1309/AAYH-1FK8-38PL-Q6DT 11939737

[29] Köksal H Dillard P Josefsson SE Maggadottir SM Pollmann S Fåne A. Preclinical development of CD37CAR T-cell therapy for treatment of B-cell lymphoma. Blood Advances. (2019) 3:1230–43. doi: 10.1182/bloodadvances.2018029678 PMC6482360 30979721

[30] Kokalaki E Ma B Ferrari M Grothier T Hazelton W Manzoor S. Dual targeting of CD19 and CD22 against B-ALL using a novel high-sensitivity aCD22 CAR. Mol Ther. (2023) 31:2089–104. doi: 10.1016/j.ymthe.2023.03.020 PMC10362402 36945773

[31] Haso W Lee DW Shah NN Stetler-Stevenson M Yuan CM Pastan IH. Anti-CD22–chimeric antigen receptors targeting B-cell precursor acute lymphoblastic leukemia. Blood. (2013) 121:1165–74. doi: 10.1182/blood-2012-06-438002 PMC3575759 23243285

[32] Fry TJ Shah NN Orentas RJ Stetler-Stevenson M Yuan CM Ramakrishna S. CD22-targeted CAR T cells induce remission in B-ALL that is naive or resistant to CD19-targeted CAR immunotherapy. Nat Med. (2018) 24:20–8. doi: 10.1038/nm.4441 PMC5774642 29155426

[33] Fergusson NJ Adeel K Kekre N Atkins H Hay KA. A systematic review and meta-analysis of CD22 CAR T-cells alone or in combination with CD19 CAR T-cells. Front Immunol. (2023) 14:1178403. doi: 10.3389/fimmu.2023.1178403 37180149 PMC10174241

[34] Baird JH Frank MJ Craig J Patel S Spiegel JY Sahaf B. CD22-directed CAR T-cell therapy induces complete remissions in CD19-directed CAR–refractory large B-cell lymphoma. Blood. (2021) 137:2321–5. doi: 10.1182/blood.2020009432 PMC8085484 33512414

[35] Frank MJ Baird J Kramer A Patel S Sahaf B Craig J. S230: CD22 CAR T cell therapy is safe and effective in patients with large B cell lymphoma who have relapsed after CD19 CAR T cell therapy. Hemasphere. (2023) 7:e3362169. doi: 10.1097/01.HS9.0000967832.33621.69

[36] Summers C Baxter B Annesley C Yokoyama J Rhea S Huang W. CD22 CAR optimization for improved in-human activity following inadequate CD22 CAR activity in phase 1 clinical trial PLAT-04. Blood. (2021) 138:403. doi: 10.1182/blood-2021-147928

[37] Johnson A Wright H Hu X Kinder J van Hoeven N Liang O. Hypoimmune, allogeneic CD22-directed CAR T cells that evade innate and adaptive immune rejection for the treatment of large B cell lymphoma patients that are relapsed/refractory to CD19-directed CAR T cell therapy. Blood. (2023) 142:3437. doi: 10.1182/blood-2023-187096

[38] Hines MR Keenan C Maron Alfaro G Cheng C Zhou Y Sharma A. Hemophagocytic lymphohistiocytosis-like toxicity (carHLH) after CD19-specific CAR T-cell therapy. Br J Haematol. (2021) 194:701–7. doi: 10.1111/bjh.17662 PMC8756350 34263927

[39] Lichtenstein DA Schischlik F Shao L Steinberg SM Yates B Wang H-W. Characterization of HLH-like manifestations as a CRS variant in patients receiving CD22 CAR T cells. Blood. (2021) 138:2469–84. doi: 10.1182/blood.2021011898 PMC8832442 34525183

[40] Jess J Yates B Dulau-Florea A Parker K Inglefield J Lichtenstein D. CD22 CAR T-cell associated hematologic toxicities, endothelial activation and relationship to neurotoxicity. J ImmunoTherapy Cancer. (2023) 11:e005898. doi: 10.1136/jitc-2022-005898 PMC10277551 37295816

[41] Kramer AM Hamilton MP Prabhu S Desai M Kuo A Ehlinger Z. Transcriptional profiling associated with CD22 CAR T cell clinical response in LBCL. Blood. (2023) 142:2091. doi: 10.1182/blood-2023-187615

[42] Hussein MS Li Q Mao R Peng Y He Y. TCR T cells overexpressing c-Jun have better functionality with improved tumor infiltration and persistence in hepatocellular carcinoma. Front Immunol. (2023) 14. doi: 10.3389/fimmu.2023.1114770 PMC10192869 37215108

[43] Shalabi H Kraft IL Wang HW Yuan CM Yates B Delbrook C. Sequential loss of tumor surface antigens following chimeric antigen receptor T-cell therapies in diffuse large B-cell lymphoma. Haematologica. (2018) 103:e215–e8. doi: 10.3324/haematol.2017.183459 PMC5927974 29419431

[44] Ramakrishna S Highfill SL Walsh Z Nguyen SM Lei H Shern JF. Modulation of target antigen density improves CAR T-cell functionality and persistence. Clin Cancer Res. (2019) 25:5329–41. doi: 10.1158/1078-0432.CCR-18-3784 PMC8290499 31110075

[45] Chen WC Completo GC Sigal DS Crocker PR Saven A Paulson JC. *In vivo* targeting of B-cell lymphoma with glycan ligands of CD22. Blood. (2010) 115:4778–86. doi: 10.1182/blood-2009-12-257386 PMC2890185 20181615

[46] Wang X Lang S Tian Y Zhang J Yan X Fang Z. Glycoengineering of natural killer cells with CD22 ligands for enhanced anticancer immunotherapy. ACS Cent Science. (2020) 6:382–9. doi: 10.1021/acscentsci.9b00956 PMC7099595 32232138

[47] Velasquez MP Szoor A Bonifant CL Vaidya A Brunetti L Gundry MC. Two-pronged cell therapy for B-cell Malignancies: engineering NK cells to target CD22 and redirect bystander T cells to CD19. Blood. (2016) 128:4560. doi: 10.1182/blood.V128.22.4560.4560

[48] Tian X Zhang R Qin H Shi X Qi W Jiang D. Immunotherapy of B cell lymphoma with CD22-redirected CAR NK-92 cells. Cent Eur J of&nbsp;Immunology. (2023) 48:1–13. doi: 10.5114/ceji.2023.126672 PMC10189576 37206593

[49] Cronk RJ Zurko J Shah NN. Bispecific chimeric antigen receptor T cell therapy for B cell Malignancies and multiple myeloma. Cancers (Basel). (2020) 12:2523. doi: 10.3390/cancers12092523 32899464 PMC7564024

[50] Schneider D Xiong Y Wu D Nölle V Schmitz S Haso W. A tandem CD19/CD20 CAR lentiviral vector drives on-target and off-target antigen modulation in leukemia cell lines. J ImmunoTherapy Cancer. (2017) 5:42. doi: 10.1186/s40425-017-0246-1 PMC5433150 28515942

[51] Shah NN Johnson BD Schneider D Zhu F Szabo A Keever-Taylor CA. Bispecific anti-CD20, anti-CD19 CAR T cells for relapsed B cell Malignancies: a phase 1 dose escalation and expansion trial. Nat Med. (2020) 26:1569–75. doi: 10.1038/s41591-020-1081-3 33020647

[52] Zurko JC Fenske TS Johnson BD Bucklan D Szabo A Xu H. Long-term outcomes and predictors of early response, late relapse, and survival for patients treated with bispecific LV20. 19 CAR T-cells Am J Hematol. (2022) 97:1580–8. doi: 10.1002/ajh.26718 36068950

[53] Shah NN Furqan F Szabo A Kearl T Zamora A Neumann J. Adaptive manufacturing of LV20.19 CAR T-cells for relapsed, refractory mantle cell lymphoma. Blood. (2023) 142:1024. doi: 10.1182/blood-2023-173954

[54] Borchmann P Lohneis A Gödel P Balke-Want H Schmid C Ayuk F. P1184: Phase I trial of MB-CART2019.1 in patientes with relapsed or refratory B-cell non-hodgkin lymphoma: 2 year follow-up report. HemaSphere. (2022) 6:1070–1. doi: 10.1097/01.HS9.0000847600.12970.7d

[55] Borchmann P Vandenberghe P Urbano-Ispizua A Haiuon C Griškevicius L Lemonnier F. A randomized phase II study of MB-CART2019.1 compared to standard of care in patients with relapsed/refractory DLBCL ineligible for ASCT – DALY 2-EU trial. Hematological Oncol. (2023) 41:840–1. doi: 10.1002/hon.3166_OT20

[56] Shah N Maziarz R Jacobson C Isufi I Ghosh M Ulrickson M. P1134: Bispecific anti-CD20/19 CAR-T – zamtocabtagene autoleucel for relapsed/refractory DLBCL – interim analysis results of DALY-II-usa study. Hemasphere. (2023) 7:e07109dc. doi: 10.1097/01.HS9.0000971432.07109.dc

[57] Tong C Zhang Y Liu Y Ji X Zhang W Guo Y. Optimized tandem CD19/CD20 CAR-engineered T cells in refractory/relapsed B-cell lymphoma. Blood. (2020) 136:1632–44. doi: 10.1182/blood.2020005278 PMC7596761 32556247

[58] Zhang Y Wang Y Liu Y Tong C Wang C Guo Y. Long-term activity of tandem CD19/CD20 CAR therapy in refractory/relapsed B-cell lymphoma: a single-arm, phase 1–2 trial. Leukemia. (2022) 36:189–96. doi: 10.1038/s41375-021-01345-8 PMC8727291 34272481

[59] Liang A Zhou L Li P Yu W Yang M Xu Y. Safety and efficacy of a novel anti-CD20/CD19 bi-specific CAR T-cell therapy (C-CAR039) in relapsed or refractory (r/r) B-cell non-Hodgkin lymphoma (B-NHL). J Clin Oncol. (2021) 39:2507. doi: 10.1200/JCO.2021.39.15_suppl.2507

[60] Li P Yu W-J Zhou L Yang M Ye S Zhu J. C-CAR039, a novel anti-CD20/CD19 bi-specific CAR T-cell therapy shows deep and durable clinical benefits in patients with relapsed or refractory (r/r) B-cell non-hodgkin lymphoma (B-NHL) in long term follow up. Blood. (2023) 142:1025. doi: 10.1182/blood-2023-182817

[61] Berger C Jensen MC Lansdorp PM Gough M Elliott C Riddell SR. Adoptive transfer of effector CD8+ T cells derived from central memory cells establishes persistent T cell memory in primates. J Clin Invest. (2008) 118:294–305. doi: 10.1172/JCI32103 18060041 PMC2104476

[62] Xu Y Zhang M Ramos CA Durett A Liu E Dakhova O. Closely related T-memory stem cells correlate with in *vivo* expansion of CAR.CD19-T cells and are preserved by IL-7 and IL-15. Blood. (2014) 123:3750–9. doi: 10.1182/blood-2014-01-552174 PMC4055922 24782509

[63] Larson SM Walthers CM Ji B Ghafouri SN Naparstek J Trent J. CD19/CD20 bispecific chimeric antigen receptor (CAR) in naive/memory T cells for the treatment of relapsed or refractory non-hodgkin lymphoma. Cancer Discovery. (2023) 13:580–97. doi: 10.1158/2159-8290.CD-22-0964 PMC9992104 36416874

[64] Good Z Spiegel JY Sahaf B Malipatlolla MB Ehlinger ZJ Kurra S. Post-infusion CAR TReg cells identify patients resistant to CD19-CAR therapy. Nat Med. (2022) 28:1860–71. doi: 10.1038/s41591-022-01960-7 PMC10917089 36097223

[65] Luo W Napoleon JV Zhang F Lee YG Wang B Putt KS. Repolarization of tumor-infiltrating myeloid cells for augmentation of CAR T cell therapies. Front Immunol. (2022) 13. doi: 10.3389/fimmu.2022.816761 PMC8889096 35250995

[66] Lam N Choi S Yang S Finney R Cam M Wu X. Development of a bicistronic anti-CD19/CD20 CAR construct including optimization to abrogate retroviral recombination events. Blood. (2021) 138:4808. doi: 10.1182/blood-2021-150865

[67] Zheng P Xue F Yang F Feng S Guo Y Shi H. P1168: Durable remission after sequential CD19/20 CAR T-cell “cake-icing” therapy occurred in refractory/relapsed DLBCL. Hemasphere. (2023) 7:e04478eb. doi: 10.1097/01.HS9.0000971568.04478.eb

[68] Cordoba S Onuoha S Thomas S Pignataro DS Hough R Ghorashian S. CAR T cells with dual targeting of CD19 and CD22 in pediatric and young adult patients with relapsed or refractory B cell acute lymphoblastic leukemia: a phase 1 trial. Nat Med. (2021) 27:1797–805. doi: 10.1038/s41591-021-01497-1 PMC8516648 34642489

[69] Dai H Wu Z Jia H Tong C Guo Y Ti D. Bispecific CAR-T cells targeting both CD19 and CD22 for therapy of adults with relapsed or refractory B cell acute lymphoblastic leukemia. J Hematol Oncol. (2020) 13:30. doi: 10.1186/s13045-020-00856-8 32245502 PMC7126394

[70] Wang Y Yang Y Hong R Zhao H Wei G Wu W. A retrospective comparison of CD19 single and CD19/CD22 bispecific targeted chimeric antigen receptor T cell therapy in patients with relapsed/refractory acute lymphoblastic leukemia. Blood Cancer J. (2020) 10:105. doi: 10.1038/s41408-020-00371-6 33077713 PMC7572410

[71] Kim H Han M Kim M Kim H Im HJ Kim N. CD19/CD22 bispecific chimeric antigen receptor−NK−92 cells are developed and evaluated. Oncol Lett. (2023) 25:236. doi: 10.3892/ol.2023.13822 37153038 PMC10161343

[72] Gardner R Annesley C Finney O Summers C Lamble AJ Rivers J. Early clinical experience of CD19 x CD22 dual specific CAR T cells for enhanced anti-leukemic targeting of acute lymphoblastic leukemia. Blood. (2018) 132:278. doi: 10.1182/blood-2018-99-113126

[73] Gardner RA Annesley C Wilson A Summers C Narayanaswamy P Wu V. Efficacy of SCRI-CAR19x22 T cell product in B-ALL and persistence of anti-CD22 activity. J Clin Oncol. (2020) 38:3035. doi: 10.1200/JCO.2020.38.15_suppl.3035

[74] Annesley C Summers C Pulsipher MA Skiles JL Li AM Vatsayan A. SCRI-CAR19x22v2 T cell product demonstrates bispecific activity in B-ALL. Blood. (2021) 138:470. doi: 10.1182/blood-2021-148881

[75] Lai X Gu X Tsao S-T Zhang Q Liu Y-C Yaxian J. Double CD19/CD22 chimeric antigen receptor-modified T cells for the treatment of stage IV relapsed and refractory follicular lymphoma. Blood. (2017) 130:5154. doi: 10.1182/blood.V130.Suppl_1.5154.5154

[76] Jiao C Zvonkov E Lai X Zhang R Liu Y Qin Y. 4SCAR2.0: a multi-CAR-T therapy regimen for the treatment of relapsed/refractory B cell lymphomas. Blood Cancer J. (2021) 11:59. doi: 10.1038/s41408-021-00455-x 33727525 PMC7966809

[77] Wang N Hu X Cao W Li C Xiao Y Cao Y. Efficacy and safety of CAR19/22 T-cell cocktail therapy in patients with refractory/relapsed B-cell Malignancies. Blood. (2020) 135:17–27. doi: 10.1182/blood.2019000017 31697824

[78] Cao Y Xiao Y Wang N Wang G Huang L Hong Z. CD19/CD22 chimeric antigen receptor T cell cocktail therapy following autologous transplantation in patients with relapsed/refractory aggressive B cell lymphomas. Transplant Cell Ther. (2021) 27:910.e1–.e11. doi: 10.1016/j.jtct.2021.08.012 34425260

[79] Roddie C Lekakis LJ Marzolini MAV Ramakrishnan A Zhang Y Hu Y. Dual targeting of CD19 and CD22 with bicistronic CAR-T cells in patients with relapsed/refractory large B-cell lymphoma. Blood. (2023) 141:2470–82. doi: 10.1182/blood.2022018598 PMC10646794 36821767

[80] Amrolia PJ Wynn R Hough RE Vora A Bonney D Veys P. Phase I study of AUTO3, a bicistronic chimeric antigen receptor (CAR) T-cell therapy targeting CD19 and CD22, in pediatric patients with relapsed/refractory B-cell acute lymphoblastic leukemia (r/r B-ALL): amelia study. Blood. (2019) 134:2620. doi: 10.1182/blood-2019-123424

[81] Abramson JS. A new CAR takes a test drive in DLBCL. Blood. (2023) 141:2410–1. doi: 10.1182/blood.2023020131 37200062

[82] Qin H Ramakrishna S Nguyen S Fountaine TJ Ponduri A Stetler-Stevenson M. Preclinical development of bivalent chimeric antigen receptors targeting both CD19 and CD22. Mol Ther - Oncolytics. (2018) 11:127–37. doi: 10.1016/j.omto.2018.10.006 PMC6300726 30581986

[83] Spiegel JY Patel S Muffly L Hossain NM Oak J Baird JH. CAR T cells with dual targeting of CD19 and CD22 in adult patients with recurrent or refractory B cell Malignancies: a phase 1 trial. Nat Med. (2021) 27:1419–31. doi: 10.1038/s41591-021-01436-0 PMC8363505 34312556

[84] Velasco-Hernandez T Zanetti SR Roca-Ho H Gutierrez-Aguera F Petazzi P Sánchez-Martínez D. Efficient elimination of primary B-ALL cells in vitro and in *vivo* using a novel 4-1BB-based CAR targeting a membrane-distal CD22 epitope. J Immunother Cancer. (2020) 8:e000896. doi: 10.1136/jitc-2020-000896 32788237 PMC7422657

[85] Zanetti SR Velasco-Hernandez T Gutierrez-Agüera F Díaz VM Romecín PA Roca-Ho H. A novel and efficient tandem CD19- and CD22-directed CAR for B cell ALL. Mol Ther. (2022) 30:550–63. doi: 10.1016/j.ymthe.2021.08.033 PMC8821938 34478871

[86] Wei G Zhang Y Zhao H Wang Y Liu Y Liang B. CD19/CD22 dual-targeted CAR T-cell therapy for relapsed/refractory aggressive B-cell lymphoma: A safety and efficacy study. Cancer Immunol Res. (2021) 9:1061–70. doi: 10.1158/2326-6066.CIR-20-0675 34290048

[87] Zhang Y Li J Lou X Chen X Yu Z Kang L. A prospective investigation of bispecific CD19/22 CAR T cell therapy in patients with relapsed or refractory B cell non-hodgkin lymphoma. Front Oncol. (2021) 11:664421. doi: 10.3389/fonc.2021.664421 34113569 PMC8185372

[88] Wang Y Tong C Dai H Wu Z Han X Guo Y. Low-dose decitabine priming endows CAR T cells with enhanced and persistent antitumor potential via epigenetic reprogramming. Nat Commun. (2021) 12:409. doi: 10.1038/s41467-020-20696-x 33462245 PMC7814040

[89] Qu C Zou R Wang P Zhu Q Kang L Ping N. Decitabine-primed tandem CD19/CD22 CAR-T therapy in relapsed/refractory diffuse large B-cell lymphoma patients. Front Immunol. (2022) 13:969660. doi: 10.3389/fimmu.2022.969660 36059523 PMC9429371

[90] Aranda-Orgilles B Chion-Sotinel I Skinner J Grudman S Mumford B Dixon C. Preclinical evidence of an allogeneic dual CD20xCD22 CAR to target a broad spectrum of patients with B-cell Malignancies. Cancer Immunol Res. (2023) 11:946–61. doi: 10.1158/2326-6066.CIR-22-0910 PMC10320475 37257169

[91] Poirot L Philip B Schiffer-Mannioui C Le Clerre D Chion-Sotinel I Derniame S. Multiplex genome-edited T-cell manufacturing platform for "Off-the-shelf" Adoptive T-cell immunotherapies. Cancer Res. (2015) 75:3853–64. doi: 10.1158/0008-5472.CAN-14-3321 26183927

[92] Abramson JS Ramakrishnan A Pierola AA Braunschweig I Cartron G Thieblemont C. Preliminary results of nathali-01: A first-in-human phase I/IIa study of UCART20x22, a dual allogeneic CAR-T cell product targeting CD20 and CD22, in relapsed or refractory (R/R) non-hodgkin lymphoma (NHL). Blood. (2023) 142:2110. doi: 10.1182/blood-2023-186570

[93] Smulski CR Eibel H. BAFF and BAFF-receptor in B cell selection and survival. Front Immunol. (2018) 9:2285. doi: 10.3389/fimmu.2018.02285 30349534 PMC6186824

[94] Novak AJ Grote DM Stenson M Ziesmer SC Witzig TE Habermann TM. Expression of BLyS and its receptors in B-cell non-Hodgkin lymphoma: correlation with disease activity and patient outcome. Blood. (2004) 104:2247–53. doi: 10.1182/blood-2004-02-0762 15251985

[95] Qin H Dong Z Wang X Cheng WA Wen F Xue W. CAR T cells targeting BAFF-R can overcome CD19 antigen loss in B cell Malignancies. Sci Trans Med. (2019) 11:eaaw9414. doi: 10.1126/scitranslmed.aaw9414 PMC7015136 31554741

[96] Wong DP Roy NK Zhang K Anukanth A Asthana A Shirkey-Son NJ. A BAFF ligand-based CAR-T cell targeting three receptors and multiple B cell cancers. Nat Commun. (2022) 13:217. doi: 10.1038/s41467-021-27853-w 35017485 PMC8752722

[97] Lemal R Tournilhac O. State-of-the-art for CAR T-cell therapy for chronic lymphocytic leukemia in 2019. J ImmunoTherapy Cancer. (2019) 7:202. doi: 10.1186/s40425-019-0686-x PMC6676603 31370892

[98] Hoffmann J-M Schubert M-L Wang L Hückelhoven A Sellner L Stock S. Differences in expansion potential of naive chimeric antigen receptor T cells from healthy donors and untreated chronic lymphocytic leukemia patients. Front Immunol. (2018) 8. doi: 10.3389/fimmu.2017.01956 PMC5767585 29375575

[99] Brudno JN Somerville RPT Shi V Rose JJ Halverson DC Fowler DH. Allogeneic T cells that express an anti-CD19 chimeric antigen receptor induce remissions of B-cell Malignancies that progress after allogeneic hematopoietic stem-cell transplantation without causing graft-versus-host disease. J Clin Oncol. (2016) 34:1112–21. doi: 10.1200/JCO.2015.64.5929 PMC4872017 26811520

[100] Todorovic Z Todorovic D Markovic V Ladjevac N Zdravkovic N Djurdjevic P. CAR T cell therapy for chronic lymphocytic leukemia: successes and shortcomings. Curr Oncol. (2022) 29:3647–57. doi: 10.3390/curroncol29050293 PMC9139644 35621683

[101] Barbanti MC Appleby N Kesavan M Eyre TA. Cellular therapy in high-risk relapsed/refractory chronic lymphocytic leukemia and richter syndrome. Front Oncol. (2022) 12:888109. doi: 10.3389/fonc.2022.888109 35574335 PMC9095984

[102] Blackmon A Danilov AV Wang L Pillai R Babaei Mirshkarlo H Rosen ST. Richter's transformation after CD-19 directed CAR-T cells for relapsed/refractory chronic lymphocytic leukemia (CLL). Blood. (2021) 138:1430. doi: 10.1182/blood-2021-149815

[103] Giordano Attianese GM Marin V Hoyos V Savoldo B Pizzitola I Tettamanti S. *In vitro* and in *vivo* model of a novel immunotherapy approach for chronic lymphocytic leukemia by anti-CD23 chimeric antigen receptor. Blood. (2011) 117:4736–45. doi: 10.1182/blood-2010-10-311845 PMC3100686 21406718

[104] Tettamanti S Rotiroti MC Giordano Attianese GMP Arcangeli S Zhang R Banerjee P. Lenalidomide enhances CD23.CAR T cell therapy in chronic lymphocytic leukemia. Leuk Lymphoma. (2022) 63:1566–79. doi: 10.1080/10428194.2022.2043299 PMC9828187 35259043

[105] Faitschuk E Hombach AA Frenzel LP Wendtner C-M Abken H. Chimeric antigen receptor T cells targeting Fc μ receptor selectively eliminate CLL cells while sparing healthy B cells. Blood. (2016) 128:1711–22. doi: 10.1182/blood-2016-01-692046 27535994

[106] Kovalovsky D Yoon JH Cyr MG Simon S Voynova E Rader C. Siglec-6 is a target for chimeric antigen receptor T-cell treatment of chronic lymphocytic leukemia. Leukemia. (2021) 35:2581–91. doi: 10.1038/s41375-021-01188-3 PMC8384967 33633313

[107] Baskar S Suschak JM Samija I Srinivasan R Childs RW Pavletic SZ. A human monoclonal antibody drug and target discovery platform for B-cell chronic lymphocytic leukemia based on allogeneic hematopoietic stem cell transplantation and phage display. Blood. (2009) 114:4494–502. doi: 10.1182/blood-2009-05-222786 PMC2777128 19667400

[108] Rankin CT Veri M-C Gorlatov S Tuaillon N Burke S Huang L. CD32B, the human inhibitory Fc-γ receptor IIB, as a target for monoclonal antibody therapy of B-cell lymphoma. Blood. (2006) 108:2384–91. doi: 10.1182/blood-2006-05-020602 16757681

[109] Lim SH Vaughan AT Ashton-Key M Williams EL Dixon SV Chan HT. Fc gamma receptor IIb on target B cells promotes rituximab internalization and reduces clinical efficacy. Blood. (2011) 118:2530–40. doi: 10.1182/blood-2011-01-330357 21768293

[110] Wang G Sun X Zuo S Li C Niu Q Xia Y. Homogeneously high expression of CD32b makes it a potential target for CAR-T therapy for chronic lymphocytic leukemia. J Hematol Oncol. (2021) 14:149. doi: 10.1186/s13045-021-01160-9 34530888 PMC8447616

[111] Farley CR Morris AB Tariq M Bennion KB Potdar S KudChadkar R. FcγRIIB is a T cell checkpoint in antitumor immunity. JCI Insight. (2021) 6:e135623. doi: 10.1172/jci.insight.135623 33616086 PMC7934918

[112] Flieswasser T Van den Eynde A Van Audenaerde J De Waele J Lardon F Riether C. The CD70-CD27 axis in oncology: the new kids on the block. J Exp Clin Cancer Res. (2022) 41:12. doi: 10.1186/s13046-021-02215-y 34991665 PMC8734249

[113] Sauer T Parikh K Sharma S Omer B Sedloev D Chen Q. CD70-specific CAR T cells have potent activity against acute myeloid leukemia without HSC toxicity. Blood. (2021) 138:318–30. doi: 10.1182/blood.2020008221 PMC8323977 34323938

[114] Cheng J Zhao Y Hu H Tang L Zeng Y Deng X. Revealing the impact of CD70 expression on the manufacture and functions of CAR-70 T-cells based on single-cell transcriptomics. Cancer Immunol Immunother. (2023) 72:3163–74. doi: 10.1007/s00262-023-03475-7 PMC10992021 37382633

[115] Tu S Zhou L Huang R Zhou X Yang J Li M. Efficacy and safety of chimeric antigen receptor T cells therapy strategy with dual targeting of CD19 and CD70 to treat relapsed/refractory diffuse large B-cell lymphoma. Blood. (2023) 142:3492. doi: 10.1182/blood-2023-186888

[116] Tu S Huang R Zhou X He Y Liang Z Wang L. Combination of CD19 and CD70 specific chimeric antigen receptor T cells achieves long-term disease-free survival in relapsed and refractory primary central nervous system diffuse large B cell lymphoma. Blood. (2018) 132:5389. doi: 10.1182/blood-2018-99-117694

[117] Choi E Chang J-W Krueger J Lahr WS Pomeroy E Walsh M. Engineering CD70-directed CAR-NK cells for the treatment of hematological and solid Malignancies. Blood. (2021) 138:1691. doi: 10.1182/blood-2021-148649 34324630

[118] Nix MA Mandal K Geng H Paranjape N Lin YT Rivera JM. Surface proteomics reveals CD72 as a target for *in vitro*-evolved nanobody-based CAR-T cells in KMT2A/MLL1-rearranged B-ALL. Cancer Discovery. (2021) 11:2032–49. doi: 10.1158/2159-8290.CD-20-0242 PMC8338785 33727310

[119] Temple WC Nix MA Naik A Izgutdina A Huang BJ Wicaksono G. Framework humanization optimizes potency of anti-CD72 nanobody CAR-T cells for B-cell Malignancies. J Immunother Cancer. (2023) 11:e006985. doi: 10.1136/jitc-2023-006985 38007238 PMC10680002

[120] Izgutdina A Temple WC Nix MA Geng H Ramos E Naik A. Affinity matured CD72 CAR-T improves efficacy versus low antigen density B-cell non-hodgkin lymphoma models. Blood. (2023) 142:2068. doi: 10.1182/blood-2023-182873

[121] Polson AG Yu S-F Elkins K Zheng B Clark S Ingle GS. Antibody-drug conjugates targeted to CD79 for the treatment of non-Hodgkin lymphoma. Blood. (2007) 110:616–23. doi: 10.1182/blood-2007-01-066704 17374736

[122] Chu F Cao J Liu J Yang H Davis TJ S-q K. Chimeric antigen receptor T cells to target CD79b in B-cell lymphomas. J ImmunoTherapy Cancer. (2023) 11:e007515. doi: 10.1136/jitc-2023-007515 PMC10680003 38007239

[123] Jiang D Tian X Bian X Zhu T Qin H Zhang R. T cells redirected against Igβ for the immunotherapy of B cell lymphoma. Leukemia. (2020) 34:821–30. doi: 10.1038/s41375-019-0607-5 31624374

[124] Locke FL Miklos DB Tees M Bees T Li A Truppel-Hartmann A. CRC-403: A Phase 1/2 Study of bbT369, a Dual CD79a and CD20 Targeting CAR T Cell Drug Product with a Gene Edit, in Relapsed and/or Refractory B Cell Non-Hodgkin's Lymphoma (NHL). Blood. (2022) 140:12716–7. doi: 10.1182/blood-2022-162604

[125] Ormhøj M Scarfò I Cabral ML Bailey SR Lorrey SJ Bouffard AA. Chimeric antigen receptor T cells targeting CD79b show efficacy in lymphoma with or without cotargeting CD19. Clin Cancer Res. (2019) 25:7046–57. doi: 10.1158/1078-0432.CCR-19-1337 PMC6891163 31439577

[126] Leung I Templeton ML Lo Y Rajan A Stull SM Garrison SM. Compromised antigen binding and signaling interfere with bispecific CD19 and CD79a chimeric antigen receptor function. Blood Advances. (2023) 7:2718–30. doi: 10.1182/bloodadvances.2022008559 PMC10275707 36469024

[127] Jahn L Hombrink P Hassan C Kester MGD van der Steen DM Hagedoorn RS. Therapeutic targeting of the BCR-associated protein CD79b in a TCR-based approach is hampered by aberrant expression of CD79b. Blood. (2015) 125:949–58. doi: 10.1182/blood-2014-07-587840 25414443

[128] Lee BK Wan Y Chin ZL Deng L Deng M Leung TM. Developing ROR1 targeting CAR-T cells against solid tumors in preclinical studies. Cancers (Basel). (2022) 14:3618. doi: 10.3390/cancers14153618 35892876 PMC9331269

[129] Peng H Nerreter T Mestermann K Wachter J Chang J Hudecek M. ROR1-targeting switchable CAR-T cells for cancer therapy. Oncogene. (2022) 41:4104–14. doi: 10.1038/s41388-022-02416-5 PMC9398970 35859167

[130] Pinilla Ibarz J Lankford A Sabzevari H Shah BD Soliman H Chavez JC. A phase1/1b dose escalation/dose expansion study of prgn-3007 ultracar-T cells in patients with advanced hematologic and solid tumor Malignancies. Blood. (2022) 140:7486–7. doi: 10.1182/blood-2022-168932

[131] Wang ML Frigault MJ Yazji S Katz Y Robinson J Breitmeyer JB. Trial-in-progress: A phase 1/2 multi-center study of onct-808, a ROR1-specific CAR T, in adult patients with relapsed/refractory aggressive B cell lymphoma. Blood. (2023) 142:4857. doi: 10.1182/blood-2023-180989

[132] Chan T Scott SP Du M Bolinger C Poortman C Shepard L. Preclinical evaluation of prgn-3007, a non-viral, multigenic, autologous ROR1 ultracar-T ® Therapy with novel mechanism of intrinsic PD-1 blockade for treatment of hematological and solid cancers. Blood. (2021) 138:1694. doi: 10.1182/blood-2021-149203

[133] Jain MD Locke FL. Seeing the light: CAR T-cell targeting of lambda-restricted B-cell lymphomas. Clin Cancer Res. (2021) 27:5736–8. doi: 10.1158/1078-0432.CCR-21-1450 34417197

[134] Vera J Savoldo B Vigouroux S Biagi E Pule M Rossig C. T lymphocytes redirected against the κ light chain of human immunoglobulin efficiently kill mature B lymphocyte-derived Malignant cells. Blood. (2006) 108:3890–7. doi: 10.1182/blood-2006-04-017061 PMC1895462 16926291

[135] Ramos CA Savoldo B Torrano V Ballard B Zhang H Dakhova O. Clinical responses with T lymphocytes targeting Malignancy-associated κ light chains. J Clin Invest. (2016) 126:2588–96. doi: 10.1172/JCI86000 PMC4922690 27270177

[136] Ranganathan R Shou P Ahn S Sun C West J Savoldo B. CAR T cells targeting human immunoglobulin light chains eradicate mature B-cell Malignancies while sparing a subset of normal B cells. Clin Cancer Res. (2021) 27:5951–60. doi: 10.1158/1078-0432.CCR-20-2754 PMC8575489 33858858

[137] Martens AWJ Rietveld JM de Boer R Peters FS Ngo A van Mil L. Redirecting T-cell activity with anti-BCMA/anti-CD3 bispecific antibodies in chronic lymphocytic leukemia and other B-cell lymphomas. Cancer Res Commun. (2022) 2:330–41. doi: 10.1158/2767-9764.CRC-22-0083 PMC9981202 36875718

[138] Zhang H Gao L Liu L Wang J Wang S Gao L. A bcma and CD19 bispecific CAR-T for relapsed and refractory multiple myeloma. Blood. (2019) 134:3147. doi: 10.1182/blood-2019-131056

[139] Roex G Campillo-Davo D Flumens D Shaw PAG Krekelbergh L De Reu H. Two for one: targeting BCMA and CD19 in B-cell Malignancies with off-the-shelf dual-CAR NK-92 cells. J Trans Med. (2022) 20:124. doi: 10.1186/s12967-022-03326-6 PMC8919645 35287669

[140] Zhou Z Han Y Pan H-B Sang C-J Shi D-L Feng C. Tri-specific CD19xCD20xCD22 VHH CAR-T cells (LCAR-AIO) eradicate antigen-heterogeneous B cell tumors, enhance expansion, and prolong persistence in preclinical *in vivo* models. Blood. (2021) 138:1700. doi: 10.1182/blood-2021-150650

[141] Schneider D Xiong Y Wu D Hu P Alabanza L Steimle B. Trispecific CD19-CD20-CD22–targeting duoCAR-T cells eliminate antigen-heterogeneous B cell tumors in preclinical models. Sci Trans Med. (2021) 13:eabc6401. doi: 10.1126/scitranslmed.abc6401 33762438

[142] Vasu S Alinari L Szuminski N Schneider D Denlinger N Chan WK. A phase I clinical trial of point-of-care manufactured fresh anti-CD19/20/22 chimeric antigen receptor T cells for treatment of relapsed or refractory lymphoid Malignancies (Non-hodgkin lymphoma, acute lymphoblastic leukemia, chronic lymphocytic leukemia, B prolymphocytic leukemia). Blood. (2022) 140:7474–5. doi: 10.1182/blood-2022-167340

[143] Tamma R Ingravallo G Gaudio F d'Amati A Masciopinto P Bellitti E. The tumor microenvironment in classic hodgkin's lymphoma in responder and no-responder patients to first line ABVD therapy. Cancers (Basel). (2023) 15:2803. doi: 10.3390/cancers15102803 37345141 PMC10216100

[144] Katsin M Dormeshkin D Meleshko A Migas A Dubovik S Konoplya N. CAR-T cell therapy for classical hodgkin lymphoma. HemaSphere. (2023) 7:e971. doi: 10.1097/HS9.0000000000000971 38026793 PMC10656097

[145] Schwarting R Gerdes J Durkop H Falini B Pileri S Stein H. BER-H2: a new anti-Ki-1 (CD30) monoclonal antibody directed at a formol- resistant epitope. Blood. (1989) 74:1678–89. doi: 10.1182/blood.V74.5.1678.1678 2477085

[146] Li Z Guo W Bai O. Mechanism of action and therapeutic targeting of CD30 molecule in lymphomas. Front Oncol. (2023) 13. doi: 10.3389/fonc.2023.1301437 PMC10767573 38188299

[147] Ramos CA Ballard B Zhang H Dakhova O Gee AP Mei Z. Clinical and immunological responses after CD30-specific chimeric antigen receptor-redirected lymphocytes. J Clin Invest. (2017) 127:3462–71. doi: 10.1172/JCI94306 PMC5669573 28805662

[148] Hombach A Heuser C Sircar R Tillmann T Diehl V Pohl C. An anti-CD30 chimeric receptor that mediates CD3-zeta-independent T-cell activation against Hodgkin's lymphoma cells in the presence of soluble CD30. Cancer Res. (1998) 58:1116–9. 9515791

[149] Ramos CA Grover NS Beaven AW Lulla PD Wu M-F Ivanova A. Anti-CD30 CAR-T cell therapy in relapsed and refractory hodgkin lymphoma. J Clin Oncol. (2020) 38:3794–804. doi: 10.1200/JCO.20.01342 PMC7655020 32701411

[150] Ahmed S Flinn IW Mei M Riedell PA Armand P Grover NS. Updated results and correlative analysis: autologous CD30.CAR-T-cell therapy in patients with relapsed or refractory classical hodgkin lymphoma (CHARIOT trial). Blood. (2022) 140:7496–7. doi: 10.1182/blood-2022-158869

[151] Wang C-M Wu Z-Q Wang Y Guo Y-L Dai H-R Wang X-H. Autologous T cells expressing CD30 chimeric antigen receptors for relapsed or refractory hodgkin lymphoma: an open-label phase I trial. Clin Cancer Res. (2017) 23:1156–66. doi: 10.1158/1078-0432.CCR-16-1365 27582488

[152] Wang D Zeng C Xu B Xu JH Wang J Jiang LJ. Anti-CD30 chimeric antigen receptor T cell therapy for relapsed/refractory CD30(+) lymphoma patients. Blood Cancer J. (2020) 10:8. doi: 10.1038/s41408-020-0274-9 31974371 PMC6978321

[153] Grover NS Savoldo B. Challenges of driving CD30-directed CAR-T cells to the clinic. BMC Cancer. (2019) 19:203. doi: 10.1186/s12885-019-5415-9 30841880 PMC6404322

[154] Marques-Piubelli ML Kim DH Medeiros LJ Lu W Khan K Gomez-Bolanos LI. CD30 expression is frequently decreased in relapsed classic Hodgkin lymphoma after anti-CD30 CAR T-cell therapy. Histopathology. (2023) 83:143–8. doi: 10.1111/his.14910 36994939

[155] Caballero Gonzalez ACC Escribà-García L Montserrat R Escudero-López E Pujol-Fernández P Ujaldón-Miró C. Phase 1 clinical trial of memory-enriched academic HSP-CAR30 for the treatment of relapsed/refractory hodgkin lymphoma and CD30+ T-cell lymphoma: clinical and biological studies. Blood. (2022) 140:405–7. doi: 10.1182/blood-2022-167504

[156] Di Stasi A De Angelis B Rooney CM Zhang L Mahendravada A Foster AE. T lymphocytes coexpressing CCR4 and a chimeric antigen receptor targeting CD30 have improved homing and antitumor activity in a Hodgkin tumor model. Blood. (2009) 113:6392–402. doi: 10.1182/blood-2009-03-209650 PMC2710932 19377047

[157] Zhang S Gu C Huang L Wu H Shi J Zhang Z. The third-generation anti-CD30 CAR T-cells specifically homing to the tumor and mediating powerful antitumor activity. Sci Rep. (2022) 12:10488. doi: 10.1038/s41598-022-14523-0 35729339 PMC9213494

[158] Grover NS Ivanova A Moore DT Cheng CJA Babinec C West J. CD30-directed CAR-T cells co-expressing CCR4 in relapsed/refractory hodgkin lymphoma and CD30+ Cutaneous T cell lymphoma. Blood. (2021) 138:742. doi: 10.1182/blood-2021-148102

[159] Voorhees TJ Beaven AW Dittus C Hucks GE Morrison JK Cheng CJA. Clinical activity of anti-PD-1 therapy following CD30 CAR-T cell therapy in relapsed hodgkin lymphoma. Blood. (2022) 140:12723–4. doi: 10.1182/blood-2022-156660

[160] Sang W Wang X Geng H Li T Li D Zhang B. Anti-PD-1 therapy enhances the efficacy of CD30-directed chimeric antigen receptor T cell therapy in patients with relapsed/refractory CD30+ Lymphoma. Front Immunol. (2022) 13. doi: 10.3389/fimmu.2022.858021 PMC9010867 35432352

[161] Zhang P Yang X Cao Y Wang J Zhou M Chen L. Autologous stem cell transplantation in tandem with Anti-CD30 CAR T-cell infusion in relapsed/refractory CD30+ lymphoma. Exp Hematol Oncol. (2022) 11:72. doi: 10.1186/s40164-022-00323-9 36253833 PMC9578248

[162] Brudno JN Natrakul DA Karrs JX Patel N Maass-Moreno R Ahlman MA. Transient responses and significant toxicities of anti-CD30 CAR T cells for CD30+ lymphomas: results of a phase I trial. Blood Adv. (2023) 8(3):802–14. doi: 10.1182/bloodadvances.2023011470 PMC10874855 37939262

[163] Svoboda J Rheingold SR Gill SI Grupp SA Lacey SF Kulikovskaya I. Nonviral RNA chimeric antigen receptor–modified T cells in patients with Hodgkin lymphoma. Blood. (2018) 132:1022–6. doi: 10.1182/blood-2018-03-837609 29925499

[164] Xue Y Lai X Li R Ge C Zeng B Li Z. CD19 and CD30 CAR T-cell immunotherapy for high-risk classical hodgkin's lymphoma. Front Oncol. (2020) 10:607362. doi: 10.3389/fonc.2020.607362 33604290 PMC7885818

[165] Tzankov A Krugmann J Fend F Fischhofer M Greil R Dirnhofer S. Prognostic significance of CD20 expression in classical hodgkin lymphoma: A clinicopathological study of 119 cases1. Clin Cancer Res. (2003) 9:1381–6. 12684408

[166] Zhao X Ma Y Bian H Liu Z. CD20 expression is closely associated with Epstein–Barr virus infection and an inferior survival in nodular sclerosis classical Hodgkin lymphoma. Front Oncol. (2022) 12. doi: 10.3389/fonc.2022.993768 PMC9486205 36147921

[167] Rehwald U Schulz H Reiser M Sieber M Staak JO Morschhauser F. Treatment of relapsed CD20+ Hodgkin lymphoma with the monoclonal antibody rituximab is effective and well tolerated: results of a phase 2 trial of the German Hodgkin Lymphoma Study Group. Blood. (2003) 101:420–4. doi: 10.1182/blood.V101.2.420 12509381

[168] El Achi H Dupont E Paul S Khoury JD. CD123 as a biomarker in hematolymphoid Malignancies: principles of detection and targeted therapies. Cancers (Basel). (2020) 12:3087. doi: 10.3390/cancers12113087 33113953 PMC7690688

[169] Ruella M Klichinsky M Kenderian SS Shestova O Ziober A Kraft DO. Overcoming the immunosuppressive tumor microenvironment of hodgkin lymphoma using chimeric antigen receptor T cells. Cancer Discovery. (2017) 7:1154–67. doi: 10.1158/2159-8290.CD-16-0850 PMC5628114 28576927

[170] Gower M Tikhonova AN. Avoiding fratricide: a T-ALL order. Blood. (2022) 140:3–4. doi: 10.1182/blood.2022016772 35797017

[171] Maciocia PM Wawrzyniecka PA Maciocia NC Burley A Karpanasamy T Devereaux S. Anti-CCR9 chimeric antigen receptor T cells for T-cell acute lymphoblastic leukemia. Blood. (2022) 140:25–37. doi: 10.1182/blood.2021013648 35507686

[172] Scarfò I Ormhøj M Frigault MJ Castano AP Lorrey S Bouffard AA. Anti-CD37 chimeric antigen receptor T cells are active against B- and T-cell lymphomas. Blood. (2018) 132:1495–506. doi: 10.1182/blood-2018-04-842708 PMC6172564 30089630

[173] Luo L Zhou X Zhou L Liang Z Yang J Tu S. Current state of CAR-T therapy for T-cell Malignancies. Ther Adv Hematol. (2022) 13:20406207221143025. doi: 10.1177/20406207221143025 36601636 PMC9806442

[174] Morris KE Bowman J Lee J-A Tyner JW Palazzolo M Druker BJ. CAR-NK specific targeting of clonal tcrvβ Chain to treat T-cell non-hodgkin's lymphoma. Blood. (2023) 142:6841. doi: 10.1182/blood-2023-189477

[175] Dimitri A Herbst F Fraietta JA. Engineering the next-generation of CAR T-cells with CRISPR-Cas9 gene editing. Mol Cancer. (2022) 21:78. doi: 10.1186/s12943-022-01559-z 35303871 PMC8932053

[176] Hill LC Rouce RH Smith TS Boriskie B Srinivasan M Thakkar SG. Enhanced anti-tumor activity of CD5 CAR T cells manufactured with tyrosine kinase inhibitors in patients with relapsed/refractory T-ALL. J Clin Oncol. (2023) 41:7002. doi: 10.1200/JCO.2023.41.16_suppl.7002

[177] Watanabe N Mo F Zheng R Ma R Bray VC van Leeuwen DG. Feasibility and preclinical efficacy of CD7-unedited CD7 CAR T cells for T cell Malignancies. Mol Ther. (2023) 31:24–34. doi: 10.1016/j.ymthe.2022.09.003 36086817 PMC9840107

[178] Li H Song W Wu J Shi Z Gao Y Li J. CAR-T cells targeting CD38 and LMP1 exhibit robust antitumor activity against NK/T cell lymphoma. BMC Med. (2023) 21:330. doi: 10.1186/s12916-023-03040-0 37649020 PMC10470138

[179] Mamonkin M Rouce RH Tashiro H Brenner MK. A T-cell-directed chimeric antigen receptor for the selective treatment of T-cell Malignancies. Blood. (2015) 126:983–92. doi: 10.1182/blood-2015-02-629527 PMC4543231 26056165

[180] Mamonkin M Mukherjee M Srinivasan M Sharma S Gomes-Silva D Mo F. Reversible transgene expression reduces fratricide and permits 4-1BB costimulation of CAR T cells directed to T-cell Malignancies. Cancer Immunol Res. (2018) 6:47–58. doi: 10.1158/2326-6066.CIR-17-0126 29079655 PMC5963729

[181] Hill LC Rouce RH Wu M Wang T Ma R Zhang H. Anti-tumor efficacy and safety of unedited autologous CD5.CAR T cells in relapsed/refractory mature T-cell lymphomas. Blood. (2023) 143(13):1231–41. doi: 10.1182/blood.2023022204 PMC10997912 38145560

[182] Pan J Tan Y Shan L Deng B Ling Z Song W. Phase I study of donor-derived CD5 CAR T cells in patients with relapsed or refractory T-cell acute lymphoblastic leukemia. J Clin Oncol. (2022) 40:7028. doi: 10.1200/JCO.2022.40.16_suppl.7028

[183] Chen KH Wada M Pinz KG Liu H Lin KW Jares A. Preclinical targeting of aggressive T-cell Malignancies using anti-CD5 chimeric antigen receptor. Leukemia. (2017) 31:2151–60. doi: 10.1038/leu.2017.8 PMC5629371 28074066

[184] Xu Y Liu Q Zhong M Wang Z Chen Z Zhang Y. 2B4 costimulatory domain enhancing cytotoxic ability of anti-CD5 chimeric antigen receptor engineered natural killer cells against T cell Malignancies. J Hematol Oncol. (2019) 12:49. doi: 10.1186/s13045-019-0732-7 31097020 PMC6524286

[185] Cooper ML Choi J Staser K Ritchey JK Devenport JM Eckardt K. An "off-the-shelf" fratricide-resistant CAR-T for the treatment of T cell hematologic Malignancies. Leukemia. (2018) 32:1970–83. doi: 10.1038/s41375-018-0065-5 PMC6102094 29483708

[186] Ghobadi A Aldoss I Maude S Wayne AS Bhojwani D Bajel A. P356: Phase 1/2 dose-escalation study of anti-CD7 allogenic CAR-T cell in relapsed or refractory(R/R) T-cell acute lymphoblastic leukemia/lymphoblastic lymphoma(T-ALL/LBL). Hemasphere. (2023) 7:e1789302. doi: 10.1097/01.HS9.0000968336.17893.02

[187] Pan J Tan Y Wang G Deng B Ling Z Song W. Donor-derived CD7 chimeric antigen receptor T cells for T-cell acute lymphoblastic leukemia: first-in-human, phase I trial. J Clin Oncol. (2021) 39:3340–51. doi: 10.1200/JCO.21.00389 34324392

[188] Zhang Y Li C Du M Jiang H Luo W Tang L. Allogenic and autologous anti-CD7 CAR-T cell therapies in relapsed or refractory T-cell Malignancies. Blood Cancer J. (2023) 13:61. doi: 10.1038/s41408-023-00822-w 37095094 PMC10125858

[189] Li Z An N Yang K Meng F Xu T Peng X. Donor CD7 chimeric antigen receptor T cell bridging to allogeneic hematopoietic stem cell transplantation for T cell hematologic Malignancy. Transplant Cell Ther. (2023) 29:167–73. doi: 10.1016/j.jtct.2022.11.013 36427783

[190] Lu P Liu Y Yang J Zhang X Yang X Wang H. Naturally selected CD7 CAR-T therapy without genetic manipulations for T-ALL/LBL: first-in-human phase 1 clinical trial. Blood. (2022) 140:321–34. doi: 10.1182/blood.2021014498 35500125

[191] You F Wang Y Jiang L Zhu X Chen D Yuan L. A novel CD7 chimeric antigen receptor-modified NK-92MI cell line targeting T-cell acute lymphoblastic leukemia. Am J Cancer Res. (2019) 9:64–78. 30755812 PMC6356925

[192] Zhu X-Y Liu X Wang X-B Wang A-Y Wang M Liu N-N. [Killing effect of A CD7 chimeric antigen receptor-modified NK-92MI cell line on CD7-positive hematological Malignant cells]. Zhongguo Shi Yan Xue Ye Xue Za Zhi. (2020) 28:1367–75. doi: 10.19746/j.cnki.issn.1009-2137.2020.04.049 32798428

[193] Dai Z Mu W Zhao Y Cheng J Lin H Ouyang K. T cells expressing CD5/CD7 bispecific chimeric antigen receptors with fully human heavy-chain-only domains mitigate tumor antigen escape. Signal Transduction Targeted Ther. (2022) 7:85. doi: 10.1038/s41392-022-00898-z PMC8948246 35332132

[194] Binder C Cvetkovski F Sellberg F Berg S Paternina Visbal H Sachs DH. CD2 immunobiology. Front Immunol. (2020) 11:1090. doi: 10.3389/fimmu.2020.01090 32582179 PMC7295915

[195] Angelos MG Patel RP Chiang Y-H Xie W Pajarillo R Shaw CE. Fratricide-resistant anti-CD2 chimeric antigen receptor T-cells with endogenous CD2 knockout are highly effective against T-cell neoplasms. Blood. (2023) 142:885. doi: 10.1182/blood-2023-181282

[196] Xiang J Devenport J Carter AJ Staser K Rettig MP Kim MY. An “Off-the-shelf” CD2 universal CAR-T therapy combined with a long-acting IL-7 for T-cell Malignancies. Blood. (2023) 142:764. doi: 10.1182/blood-2023-181087 PMC10681896 37798328

[197] Geng JJ Tang J Yang XM Chen R Zhang Y Zhang K. Targeting CD147 for T to NK lineage reprogramming and tumor therapy. EBioMedicine. (2017) 20:98–108. doi: 10.1016/j.ebiom.2017.05.022 28571672 PMC5478251

[198] Zhang R-Y Wei D Liu Z-K Yong Y-L Wei W Zhang Z-Y. Doxycycline inducible chimeric antigen receptor T cells targeting CD147 for hepatocellular carcinoma therapy. Front Cell Dev Biol. (2019) 7. doi: 10.3389/fcell.2019.00233 PMC6798074 31681766

[199] Chen XH Chen R Shi MY Tian RF Zhang H Xin ZQ. Chimeric antigen receptor T cells targeting CD147 for non-small cell lung cancer therapy. Transl Oncol. (2022) 16:101309. doi: 10.1016/j.tranon.2021.101309 34896852 PMC8681039

[200] Tseng H-c Xiong W Badeti S Yang Y Ma M Liu T. Efficacy of anti-CD147 chimeric antigen receptors targeting hepatocellular carcinoma. Nat Commun. (2020) 11:4810. doi: 10.1038/s41467-020-18444-2 32968061 PMC7511348

[201] Zheng NS Zhao XY Wei D Miao JL Liu ZK Yong YL. CD147-specific chimeric antigen receptor T cells effectively inhibit T cell acute lymphoblastic leukemia. Cancer Lett. (2022) 542:215762. doi: 10.1016/j.canlet.2022.215762 35659513

[202] Zhang C Palashati H Rong Z Lin N Shen L Liu Y. Pre-depletion of TRBC1+ T cells promotes the therapeutic efficacy of anti-TRBC1 CAR-T for T-cell Malignancies. Mol Cancer. (2020) 19:162. doi: 10.1186/s12943-020-01282-7 33218364 PMC7679992

[203] Cwynarski K Iacoboni G Tholouli E Menne TF Irvine DA Balasubramaniam N. First in human study of AUTO4, a TRBC1-targeting CAR T-cell therapy in relapsed/refractory TRBC1-positive peripheral T-cell lymphoma. Blood. (2022) 140:10316–7. doi: 10.1182/blood-2022-165971

[204] Maciocia PM Wawrzyniecka PA Philip B Ricciardelli I Akarca AU Onuoha SC. Targeting the T cell receptor β-chain constant region for immunotherapy of T cell Malignancies. Nat Med. (2017) 23:1416–23. doi: 10.1038/nm.4444 29131157

[205] Cwynarski K Iacoboni G Tholouili E Menne T Irvine D Balasubramaniam N. First in human study of auto4, a TRBC1-tragetting CART T cell therapy in relapsed/refractory TRBC1-positive peripheral T-cell lymphoma. Hematological Oncol. (2023) 41:80–1. doi: 10.1002/hon.3163_44

[206] Li F Zhang H Wang W Yang P Huang Y Zhang J. T cell receptor β-chain-targeting chimeric antigen receptor T cells against T cell Malignancies. Nat Commun. (2022) 13:4334. doi: 10.1038/s41467-022-32092-8 35882880 PMC9325690

[207] Safarzadeh Kozani P Safarzadeh Kozani P Rahbarizadeh F. CAR-T cell therapy in T-cell Malignancies: Is success a low-hanging fruit? Stem Cell Res Ther. (2021) 12:527. doi: 10.3389/fimmu.2021.765097 34620233 PMC8499460

[208] Rasaiyaah J Georgiadis C Preece R Mock U Qasim W. TCRαβ/CD3 disruption enables CD3-specific antileukemic T cell immunotherapy. JCI Insight. (2018) 3:e99442. doi: 10.1172/jci.insight.99442 29997304 PMC6124532

[209] Chen KH Wada M Firor AE Pinz KG Jares A Liu H. Novel anti-CD3 chimeric antigen receptor targeting of aggressive T cell Malignancies. Oncotarget. (2016) 7:56219–32. doi: 10.18632/oncotarget.11019 PMC5302909 27494836

[210] Qian H Gay FPH Pang JWL Lee Y Ang J Tan HC. Development of anti-CD3 chimeric antigen receptor (CAR)-T cells for allogeneic cell therapy of peripheral T-cell lymphoma (PTCL). Blood. (2022) 140:4510–1. doi: 10.1182/blood-2022-162222

[211] Zhang H Feng J Zhang W Chen Q Cao Y Pinz K. First-in-human CD4 CAR clinical trial on peripheral T-cell lymphoma. Blood. (2019) 134:2881. doi: 10.1182/blood-2019-122789

[212] Pinz KG Yakaboski E Jares A Liu H Firor AE Chen KH. Targeting T-cell Malignancies using anti-CD4 CAR NK-92 cells. Oncotarget. (2017) 8:112783–96. doi: 10.18632/oncotarget.22626 PMC5762550 29348865

[213] Horwitz S O'Connor OA Pro B Trümper L Iyer S Advani R. The ECHELON-2 Trial: 5-year results of a randomized, phase III study of brentuximab vedotin with chemotherapy for CD30-positive peripheral T-cell lymphoma. Ann Oncol. (2022) 33:288–98. doi: 10.1016/j.annonc.2021.12.002 PMC9447792 34921960

[214] Voorhees TJ Ghosh N Grover N Block J Cheng C Morrison K. Long-term remission in multiply relapsed enteropathy-associated T-cell lymphoma following CD30 CAR T-cell therapy. Blood Advances. (2020) 4:5925–8. doi: 10.1182/bloodadvances.2020003218 PMC7724914 33259598

[215] Frazao A Rethacker L Messaoudene M Avril M-F Toubert A Dulphy N. NKG2D/NKG2-ligand pathway offers new opportunities in cancer treatment. Front Immunol. (2019) 10. doi: 10.3389/fimmu.2019.00661 PMC6449444 30984204

[216] Spear P Barber A Rynda-Apple A Sentman CL. NKG2D CAR T-cell therapy inhibits the growth of NKG2D ligand heterogeneous tumors. Immunol Cell Biol. (2013) 91:435–40. doi: 10.1038/icb.2013.17 PMC3700668 23628805

[217] Curio S Jonsson G Marinović S. A summary of current NKG2D-based CAR clinical trials. Immunother Adv. (2021) 1:ltab018. doi: 10.1093/immadv/ltab018 34604863 PMC8480431

[218] Perera LP Zhang M Nakagawa M Petrus MN Maeda M Kadin ME. Chimeric antigen receptor modified T cells that target chemokine receptor CCR4 as a therapeutic modality for T-cell Malignancies. Am J Hematol. (2017) 92:892–901. doi: 10.1002/ajh.24794 28543380 PMC5546946

[219] Watanabe K Gomez AM Kuramitsu S Siurala M Da T Agarwal S. Identifying highly active anti-CCR4 CAR T cells for the treatment of T-cell lymphoma. Blood Adv. (2023) 7:3416–30. doi: 10.1182/bloodadvances.2022008327 PMC10345856 37058474

[220] Bobrowicz M Kusowska A Krawczyk M Slusarczyk A Barankiewicz J Domagala J. CD20 expression regulates CD37 levels in B-cell lymphoma – implications for immunotherapies. bioRxiv. (2023). doi: 10.1101/2023.12.06.570441 PMC11155707 38846084

[221] Khopanlert W Prompat N Saetang J Maneechai K Okuno S Terakura S. Impact of glycine-serine linker on target antigen binding and subsequent CD37CAR-T performance. Blood. (2023) 142:3623. doi: 10.1182/blood-2023-173354 PMC13163721 42123690

[222] Frigault MJ Chen Y-B Gallagher KME Horick NK El-Jawahri A Scarfò I. Phase 1 study of CD37-directed CAR T cells in patients with relapsed or refractory CD37+ Hematologic Malignancies. Blood. (2021) 138:653. doi: 10.1182/blood-2021-146236

[223] Imai K Takeuchi Y Terakura S Okuno S Adachi Y Osaki M. Dual CAR-T cells targeting CD19 and CD37 are effective in target antigen loss B-cell tumor models. Mol Cancer Ther. (2023) 23(3):OF1–OF13. doi: 10.1158/1535-7163.MCT-23-0408 37828726

[224] Golubovskaya V Zhou H Li F Valentine M Sun J Berahovich R. Novel CD37, humanized CD37 and bi-specific humanized CD37-CD19 CAR-T cells specifically target lymphoma. Cancers. (2021) 13:981. doi: 10.3390/cancers13050981 33652767 PMC7956426

[225] Glisovic-Aplenc T Diorio C Chukinas JA Veliz K Shestova O Shen F. CD38 as a pan-hematologic target for chimeric antigen receptor T cells. Blood Advances. (2023) 7:4418–30. doi: 10.1182/bloodadvances.2022007059 PMC10440474 37171449

[226] Isabelle C McConnell K Boles AE Brammer JE Berge R Portocarrero C. Therapeutic potential and role of CD38 in cutaneous T-cell lymphoma pathogenesis. Blood. (2022) 140:9216–8. doi: 10.1182/blood-2022-170550

[227] Johnson WT Isabelle C Vogel AN Brammer JE Boles AE McConnell K. Efficient targeting of CD38 in mature T-cell neoplasms with daratumumab and allogeneic NK cells. Blood. (2021) 138:2408. doi: 10.1182/blood-2021-154234 34324649

[228] Wang X Yu X Li W Neeli P Liu M Li L. Expanding anti-CD38 immunotherapy for lymphoid Malignancies. J Exp Clin Cancer Res. (2022) 41:210. doi: 10.1186/s13046-022-02421-2 35765110 PMC9237984

[229] Troy EC Sezgin Y Pereira MDS Caporale J Behbehani GK Lyberger J. CD38 KO/CD38-CAR human primary natural killer cells enhance cytotoxicity against CD38-expressing primary lymphoma, leukemia, and myeloma. Blood. (2023) 142:3443. doi: 10.1182/blood-2023-174742

[230] Isabelle C Boles A McConnell K Keller R Cheslow L Xu J. Pathobiology and targeting of CD38 in cutaneous T-cell lymphoma. Blood. (2023) 142:1650. doi: 10.1182/blood-2023-189556

[231] Gurney M Stikvoort A Nolan E Kirkham-McCarthy L Khoruzhenko S Shivakumar R. CD38 knockout natural killer cells expressing an affinity optimized CD38 chimeric antigen receptor successfully target acute myeloid leukemia with reduced effector cell fratricide. Haematologica. (2022) 107:437–45. doi: 10.3324/haematol.2020.271908 PMC8804573 33375774

[232] Ambinder RF. Epstein-barr virus and hodgkin lymphoma. Hematology. (2007) 2007:204–9. doi: 10.1182/asheducation-2007.1.204 18024631

[233] Donzel M Bonjour M Combes J-D Broussais F Sesques P Traverse-Glehen A. Lymphomas associated with Epstein-Barr virus infection in 2020: Results from a large, unselected case series in France. eClinicalMedicine. (2022) 54:101674. doi: 10.1016/j.eclinm.2022.101674 36204003 PMC9531037

[234] Zhang X Zhang R Ren C Xu Y Wu S Meng C. Epstein Barr virus-positive B-cell lymphoma is highly vulnerable to MDM2 inhibitors in vivo. Blood Adv. (2022) 6:891–901. doi: 10.1182/bloodadvances.2021006156 34861697 PMC8945299

[235] Murray PG Young LS. An etiological role for the Epstein-Barr virus in the pathogenesis of classical Hodgkin lymphoma. Blood. (2019) 134:591–6. doi: 10.1182/blood.2019000568 31186275

[236] Singh DR Nelson SE Pawelski AS Kansra AS Fogarty SA Bristol JA. Epstein–Barr virus LMP1 protein promotes proliferation and inhibits differentiation of epithelial cells via activation of YAP and TAZ. Proc Natl Acad Sci. (2023) 120:e2219755120. doi: 10.1073/pnas.2219755120 37155846 PMC10193989

[237] Ho C Ruella M Levine BL Svoboda J. Adoptive T-cell therapy for Hodgkin lymphoma. Blood Adv. (2021) 5:4291–302. doi: 10.1182/bloodadvances.2021005304 PMC8945637 34610100

[238] Tang X Zhou Y Li W Tang Q Chen R Zhu J. T cells expressing a LMP1-specific chimeric antigen receptor mediate antitumor effects against LMP1-positive nasopharyngeal carcinoma cells in *vitro* and in vivo. J BioMed Res. (2014) 28:468–75. doi: 10.7555/JBR.28.20140066 PMC4250525 25469116

[239] Harada S Ando M Ando J Ishii M Yamaguchi T Yamazaki S. Dual-antigen targeted iPSC-derived chimeric antigen receptor-T cell therapy for refractory lymphoma. Mol Ther. (2022) 30:534–49. doi: 10.1016/j.ymthe.2021.10.006 PMC8821952 34628050

[240] Slabik C Kalbarczyk M Danisch S Zeidler R Klawonn F Volk V. CAR-T cells targeting epstein-barr virus gp350 validated in a humanized mouse model of EBV infection and lymphoproliferative disease. Mol Ther - Oncolytics. (2020) 18:504–24. doi: 10.1016/j.omto.2020.08.005 PMC7479496 32953984

[241] Zhang AQ Hostetler A Chen LE Mukkamala V Abraham W Padilla LT. Universal redirection of CAR T cells against solid tumors via membrane-inserted ligands for the CAR. Nat Biomed Engineering. (2023) 7:1113–28. doi: 10.1038/s41551-023-01048-8 PMC10504084 37291434

[242] Aalipour A Le Boeuf F Tang M Murty S Simonetta F Lozano AX. Viral delivery of CAR targets to solid tumors enables effective cell therapy. Mol Ther - Oncolytics. (2020) 17:232–40. doi: 10.1016/j.omto.2020.03.018 PMC7183102 32346612

[243] Lai J Mardiana S House IG Sek K Henderson MA Giuffrida L. Adoptive cellular therapy with T cells expressing the dendritic cell growth factor Flt3L drives epitope spreading and antitumor immunity. Nat Immunol. (2020) 21:914–26. doi: 10.1038/s41590-020-0676-7 32424363

[244] D'Aloia MM Caratelli S Palumbo C Battella S Arriga R Lauro D. T lymphocytes engineered to express a CD16-chimeric antigen receptor redirect T-cell immune responses against immunoglobulin G–opsonized target cells. Cytotherapy. (2016) 18:278–90. doi: 10.1016/j.jcyt.2015.10.014 26705740

[245] Caratelli S Sconocchia T Arriga R Coppola A Lanzilli G Lauro D. FCγ Chimeric receptor-engineered T cells: methodology, advantages, limitations, and clinical relevance. Front Immunol. (2017) 8. doi: 10.3389/fimmu.2017.00457 PMC5406408 28496440

[246] Zahavi D Weiner L. Monoclonal antibodies in cancer therapy. Antibodies (Basel). (2020) 9:34. doi: 10.3390/antib9030034 32698317 PMC7551545

[247] Munoz J Flinn IW Cohen JB Sachs J Exter B Ranger A. Results from a phase 1 study of ACTR707 in combination with rituximab in patients with relapsed or refractory CD20(+) B cell lymphoma. Transplant Cell Ther. (2023). doi: 10.1016/j.jtct.2023.10.014 37898374

[248] Daei Sorkhabi A Mohamed Khosroshahi L Sarkesh A Mardi A Aghebati-Maleki A Aghebati-Maleki L. The current landscape of CAR T-cell therapy for solid tumors: Mechanisms, research progress, challenges, and counterstrategies. Front Immunol. (2023) 14. doi: 10.3389/fimmu.2023.1113882 PMC10067596 37020537

[249] George P Dasyam N Giunti G Mester B Bauer E Andrews B. Third-generation anti-CD19 chimeric antigen receptor T-cells incorporating a TLR2 domain for relapsed or refractory B-cell lymphoma: a phase I clinical trial protocol (ENABLE). BMJ Open. (2020) 10:e034629. doi: 10.1136/bmjopen-2019-034629 PMC7044946 32041862

[250] Svoboda J Gerson JN Landsburg DJ Chong EA Barta SK Dwivedy Nasta S. Interleukin-18 secreting autologous anti-CD19 CAR T-cells (huCART19-IL18) in patients with non-hodgkin lymphomas relapsed or refractory to prior CAR T-cell therapy. Blood. (2022) 140:4612–4. doi: 10.1182/blood-2022-162393

[251] Adachi K Kano Y Nagai T Okuyama N Sakoda Y Tamada K. IL-7 and CCL19 expression in CAR-T cells improves immune cell infiltration and CAR-T cell survival in the tumor. Nat Biotechnol. (2018) 36:346–51. doi: 10.1038/nbt.4086 29505028

[252] Perales M-A Ahmed S Dahiya S Riedell PA McGuirk JP Oluwole OO. A phase 2/3, randomized, double blind, placebo-controlled, multicenter study of NKTR-255 vs placebo following CD-19 directed CAR-T therapy in patients with relapsed/refractory large B-cell lymphoma. Blood. (2022) 140:7488–90. doi: 10.1182/blood-2022-162511

[253] Wang X Diamond DJ Forman SJ Nakamura R. Development of CMV-CD19 bi-specific CAR T cells with post-infusion in *vivo* boost using an anti-CMV vaccine. Int J Hematol. (2021) 114:544–53. doi: 10.1007/s12185-021-03215-6 PMC8475363 34561840

[254] Rataj F Kraus FBT Chaloupka M Grassmann S Heise C Cadilha BL. PD1-CD28 fusion protein enables CD4+ T cell help for adoptive T cell therapy in models of pancreatic cancer and non-hodgkin lymphoma. Front Immunol. (2018) 9:1955. doi: 10.3389/fimmu.2018.01955 30214445 PMC6125378

[255] Prosser ME Brown CE Shami AF Forman SJ Jensen MC. Tumor PD-L1 co-stimulates primary human CD8(+) cytotoxic T cells modified to express a PD1:CD28 chimeric receptor. Mol Immunol. (2012) 51:263–72. doi: 10.1016/j.molimm.2012.03.023 22503210

[256] Ankri C Shamalov K Horovitz-Fried M Mauer S Cohen CJ. Human T cells engineered to express a programmed death 1/28 costimulatory retargeting molecule display enhanced antitumor activity. J Immunol. (2013) 191:4121–9. doi: 10.4049/jimmunol.1203085 24026081

[257] Juillerat A Tkach D Busser BW Temburni S Valton J Duclert A. Modulation of chimeric antigen receptor surface expression by a small molecule switch. BMC Biotechnol. (2019) 19:44. doi: 10.1186/s12896-019-0537-3 31269942 PMC6610870

[258] Dwivedi A Karulkar A Ghosh S Srinivasan S Kumbhar BV Jaiswal AK. Robust antitumor activity and low cytokine production by novel humanized anti-CD19 CAR T cells. Mol Cancer Ther. (2021) 20:846–58. doi: 10.1158/1535-7163.MCT-20-0476 PMC12456031 33632869

[259] Karulkar A Jain H Shah S Khan A Jaiswal A Firfiray A. Making anti-CD19 CAR-T cell therapy accessible and affordable: first-in-human phase I clinical trial experience from India. Blood. (2022) 140:4610–1. doi: 10.1182/blood-2022-168928

